# Explainable AI reveals the allosteric blind spot in protein-ligand binding predictions

**DOI:** 10.1016/j.xcrp.2026.103448

**Published:** 2026-07-22

**Authors:** Vedant Parikh, Brandon Foley, Will Gatlin, Max Ludwick, Lucas Turano, Gennady M. Verkhivker

**Affiliations:** 1 Keck Center for Science and Engineering, Graduate Program in Computational and Data Sciences, Schmid College of Science and Technology, Chapman University, Orange, CA 92866, USA; 2 Department of Biomedical and Pharmaceutical Sciences, Chapman University School of Pharmacy, Irvine, CA 92618, USA; 3 Department of Pharmacology, Skaggs School of Pharmacy and Pharmaceutical Sciences, University of California, San Diego, 9500 Gilman Drive, La Jolla, CA 92093, USA; 4 Lead contact

## Abstract

Artificial intelligence (AI) has transformed prediction of protein structure and interactions, yet modeling of allosteric binding remains a persistent challenge. We develop an explainable AI framework that interrogates AI models AlphaFold3, Protenix, Boltz-2, Chai-1, and DynamicBind on rigorously stratified datasets of orthosteric and allosteric ligand-protein complexes. While AI models excel in accurate modeling of orthosteric ligand binding, a consistent and substantial performance gap observed across diverse architectures emerges in prediction of allosteric complexes. The biophysical logic for this dichotomy is unveiled through physics-based lens of the energy landscape theory. Orthosteric binding creates dominant energetic funnels via ligand-induced minimal frustration quenching, while allosteric sites preserve neutral frustration landscapes in both apo and holo protein states. By linking prediction outcomes to frustration landscapes, this study recasts AI shortcomings in prediction of allosteric ligand binding as diagnostic indicators of allostery, establishing a physics-informed framework that turns the allosteric blind spot into mechanistic insight.

## In brief

Parikh et al. reveal that AI models exhibit universal limitations in predicting allosteric ligand binding. Integrating AI predictions with the energy-landscape apparatus establishes that neutral frustration of allosteric binding creates persistent blind spots. Physics-based explainable AI reveals how protein energy landscapes govern AI performance, unveiling the biophysical code of allostery.

## Graphical Abstract

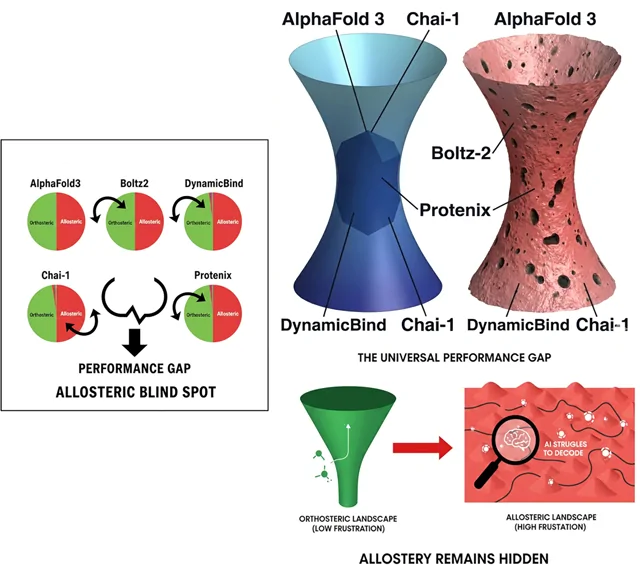

## INTRODUCTION

AI has transformed structural biology, enabling atomic-resolution modeling of biomolecular systems and accelerating therapeutic discovery.^1–5^ This revolution began with AlphaFold2 near-experimental protein structure prediction,^1,2^ extending to multi-chain complexes,^6^ sequence-based protein language models (PLMs) ESMFold^7,8^ and RoseTTAFold2,^9,10^ and generative design frameworks.^11^ Despite these advances, reliable predictions of functional allosteric sites remains a critical limitation.^12^ Current AI models excel at recovering thermodynamically stable ground states of proteins but systematically fail to accurately reproduce conformational ensembles and detect hidden allosteric states.^13–17^ Efforts to overcome this bias through multiple sequence alignment subsampling,^13^ *in silico* mutagenesis,^14^ and other sampling strategies^18–25^ have seen only partial success, constrained by training data and evolutionary priors. Concurrently, biomolecular binding prediction approaches have been shifting from purely physics-based molecular docking tools to end-to-end AI co-folding strategies. Diffusion-based architectures now jointly model protein folding and ligand placement. GeoDiff pioneered geometric denoising,^26^ TorsionDiff constrained sampling to rotatable bonds,^27^ and DiffDock achieved near-experimental ligand-pose accuracy in modeling of ligand-protein complexes^28^ and is further refined by implementation in its successor DiffDock-L conditional diffusion architecture.^29^ AlphaFold3 (AF3) unified this paradigm by initiating prediction from an atomic “cloud” to synchronize folding and docking across biomolecules.^30^ On the PoseBusters benchmark dataset,^31^ AF3 achieved 76%–81% success in recovering native ligand poses within ~2.0 Å root-mean-square deviation (RMSD), substantially outperforming classical docking tools such as AutoDock Vina^32^ (52%–60%) and DiffDock (~38%).^33^ Protenix, an open-source reproduction with architectural optimizations, employs unified tokenization of polymers and small molecules^34^ and attains comparable or slightly higher success (~80%) on PoseBusters V2.^33^ Boltz-2 extends all-atom co-folding with affinity and binder-classification heads trained on experimental and molecular dynamics (MD) simulations-derived data,^35^ achieving ~75%–81% success in blind docking^33^ and leading the CASP16 Affinity Challenge.^36,37^ Chai-1 integrates paired attention with PLM embeddings to enable accurate predictions from sparse evolutionary data while supporting flexible loop and ligand modeling via atomic diffusion.^38^ DynamicBind advances beyond static co-folding by explicitly modeling ligand-induced protein rearrangements using an SE(3)-equivariant diffusion framework, enabling cryptic pocket formation and large-scale conformational morphing.^39^

Because sequence-based models lack explicit 3D awareness, recent advances increasingly fuse PLM embeddings with structural graphs. The Equi approach^40^ and related approach DiffSBDD^41^ use equivariant graph neural networks (GNNs) to predict ligand-binding poses and pocket locations directly from apo protein structures. The TANKBind approach segments proteins into functional blocks and predicts their interactions with the ligand using a trigonometry-aware architecture.^42^ Co-folding and binding AI models can now integrate protein and ligand co-design. The RFdiffusion model^11^ and deep-learning (DL) protein sequence design method ProteinMPNN^43^ use a protein backbone diffusion model and enable *de novo* design of protein binders and small-molecule scaffolds that contain a wide range of ligand-binding pockets. A multimodal foundation model, ESM3, integrates sequences, structures, and functional annotations into a single architecture capable of zero-shot ligand generation.^44^ Chroma, developed by Generate Biomedicines, samples novel protein structures conditioned on desired properties.^45^

Parallel to co-folding advances, computational predictions of druggable binding sites expanded into a broad methodological ecosystem that encompasses geometric methods (Fpocket,^46^ SiteHound,^47^ CurPocket,^48^ and MetaPocket 2.0^49^), machine-learning (ML) tools such as P2Rank^50,51^ and ISMBLab-LIG^52^ that use 3D probability density maps of interacting atoms to identify binding sites, DL architectures (DeepSite,^53^ FRSite,^54^ BiteNet^55^), and a spectrum of PLM-based approaches^56–61^ that offer a scalable, alignment-driven framework for proteome-wide binding-site annotation, with particular strength in identifying conserved functional regions. Despite this progress, robust detection of functional allosteric and cryptic binding sites remains elusive as most methods are optimized for stable, evolutionarily conserved orthosteric pockets, whereas allosteric sites are often transient, weakly conserved, and sparsely populated in structural databases.^62^ Complementary physics-based approaches AlloPred,^63^ Allosite,^64^ AllositePro,^65^ and DeepAllo^66,67^ underscored the need to embed biophysical principles within DL prediction frameworks. While featuring high performance on ligand binding to orthosteric sites, all methods exhibit a pronounced and consistent drop in accuracy for allosteric binding sites and transient pockets that only form upon ligand binding.^68^ A recent investigation integrated local binding geometry, coevolution, and dynamic allostery into a three-parameter model to identify potentially hidden allosteric sites in ensembles of protein structures with orthosteric ligands.^69^

A similar divergence emerged in structural predictions of ligand-protein complexes and reconstruction of binding poses for orthosteric and allosteric ligands. While AI models typically excel in accurate prediction of orthosteric ligand binding, a marked performance gap is often observed across diverse architectures that emerges in prediction of allosteric complexes. In a recent study, AI co-folding methods NeuralPLexer, RoseTTAFold All-Atom and Boltz-1/Boltz-1x were used to predict the binding modes of orthosteric and allosteric small-molecule modulators using a curated dataset of 17 protein structures with orthosteric-allosteric ligand sets.^70^ The results showed thar robust prediction of ligand poses for orthosteric ligands are contrasted with consistent mispredictions of bound conformations for the allosteric ligands often placed far away from their crystallographic binding modes for all co-folding methods.

The duality in detection of canonical binding sites and functional allosteric pockets and similar emerging dichotomy in prediction of orthosteric and allosteric ligand-binding modes across a wide range of protein systems raises several important questions: (1) is the emerging divergence in prediction of allosteric binding sites and allosteric ligand-binding poses largely determined by structural memorization patterns dominated by canonical binding, or (2) can this dichotomy may be intrinsically governed by physical and evolutionary principles underlying design of allosteric sites ? Finally, how can we leverage these principles to build more interpretable and robust predictive models? In this study, we focus on a well-defined but critical task: ligand-binding mode prediction for orthosteric and allosteric sites using AI co-folding models. We do not address the broader problem of predicting allosteric regulation, long-range conformational changes, or signal propagation. Instead, we ask, given a known allosteric binding site (defined by a co-crystal structure), can current models accurately place the ligand?

The central hypothesis put forward in the current study is that the emerging limitations in AI modeling of allosteric ligand binding may reflect not only algorithmic shortcomings of current AI co-folding architectures but rather represent a more fundamental biophysical mismatch between orthosteric and allosteric binding landscapes. According to this conjecture, orthosteric sites feature strong evolutionary conservation, geometric recurrence, and pronounced energetic stabilization, which are signals perfectly aligned with inductive biases of AI models. Allosteric sites, by contrast, often reside in evolutionarily permissive, conformationally adaptive regions. Their functional versatility arises from the absence of dominant structural or energetic patterns that may often render them invisible to pattern-recognition AI models trained on recurrent motifs. These distinctions necessitate a shift to explainable biophysical AI frameworks grounded in the protein energy landscapes. A particularly informative biophysical lens is offered by local frustration analysis.^71–80^ Quantifying competing interactions within folded structures, local frustration analysis quantifies the energetic optimality of native interactions by comparing them to decoy ensembles distinguishing minimally frustrated regions optimized for stability from neutral and highly frustrated patches that are associated with conformational plasticity and functional adaptability.^72–76^ Integrating this analysis with predictive benchmarking and systematic interrogation of AI co-folding models in prediction of orthosteric and allosteric ligand binding transforms AI prediction metrics into reporters of the underlying biophysical grammar. Our analysis confirms a significant and consistent performance gap identifying the allosteric blind spot as a universal property of current architectures. We demonstrate that this divide is intrinsically linked to the distinct landscape hierarchy where orthosteric ligand-binding regions are enriched in minimally frustrated, evolutionarily constrained residues, whereas allosteric ligand binding navigates through conformationally diverse and evolutionary promiscuous neutrally frustrated regions. This study demonstrates that dichotomy in prediction of orthosteric and allosteric ligand binding arises from a principled mismatch between model inductive memorization biases of AI models and the energetic logic of allosteric regulation, therefore transforming AI uncertainties into diagnostic indicators of the elusive biophysical code underlying allosteric mechanisms.

## RESULTS

### A dual framework: AI benchmarking and explainable energy-landscape profiling

**Figure 1.**
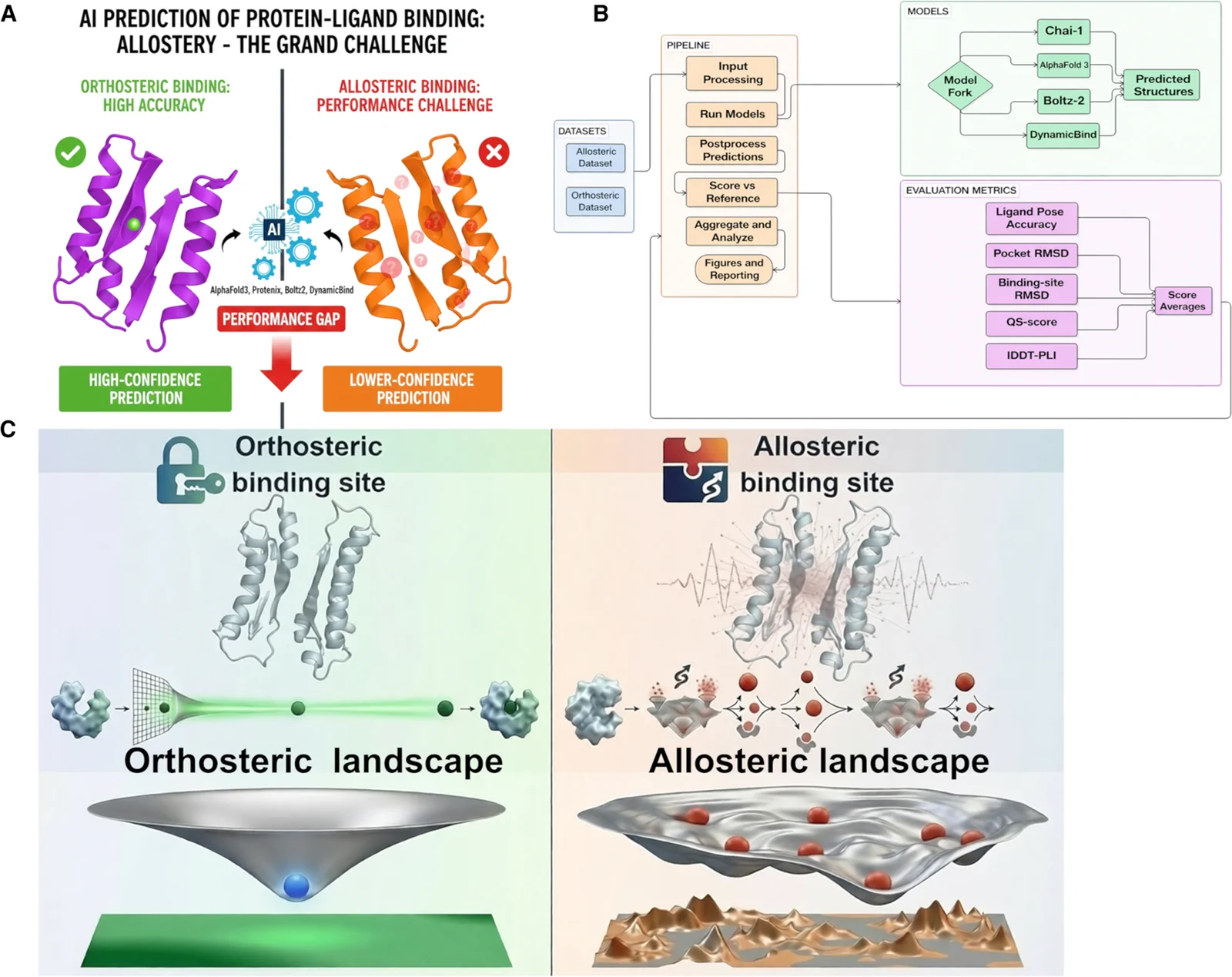
Dual explainable-AI framework for benchmarking ligand-protein binding predictions across orthosteric and allosteric sites (A) Schematic overview of the explainable-AI framework. Input consists of rigorously stratified orthosteric and allosteric datasets (left). Five state-of-the-art AI co-folding models (AF3, Protenix, Boltz-2, Chai-1, DynamicBind) are executed to produce ligand pose predictions. Outputs are evaluated using geometric metrics (ligand RMSD, pocket RMSD, quaternary structure [QS] score) and overlaid with energy-landscape analysis based on local frustration (right). The framework transforms prediction accuracy into a reporter of the underlying biophysical grammar. (B) Benchmarking pipeline details: protein sequences and ligand SMILES are fed to each model; 25 independent predictions are generated per complex; predictions are superimposed onto experimental reference structures; ligand-pose RMSD, pocket RMSD, QS score, and success rates are computed; aggregate statistical analysis is performed across datasets and models. (C) Conceptual energy landscapes contrasting orthosteric (left) and allosteric (right) binding. The vertical axis represents free energy; the horizontal axes represent conformational degrees of freedom. Left (orthosteric): the landscape is dominated by a single, deep, steep-walled funnel that narrows toward a unique global minimum corresponding to the native ligand pose. This funnel arises from ligand-induced frustration quenching (transition of binding-site residues from highly frustrated to minimally frustrated upon binding). The steep directional gradient forces conformational sampling toward a single well-defined geometry, explaining why all five models produce unimodal, low-RMSD predictions. Right (allosteric): the landscape is flat and degenerate, characterized by multiple shallow minima of comparable depth separated by low barriers. No single funnel directs sampling toward a unique pose; many geometrically distinct ligand arrangements have similar interaction topologies. The schematic is simplified to highlight the essential difference in funnel depth and uniqueness for orthosteric vs. allosteric binding.

To systematically interrogate the performance of leading AI co-folding models in prediction of orthosteric and allosteric ligand binding, we developed a dual explainable-AI framework designed to distinguish between limitations imposed by training data and those imposed by the physical organization of the binding energy landscapes (Figure 1). We systematically evaluate five state-of-the-art AI co-folding models (AF3,^30^ Protenix,^34^ Boltz-2,^35^ Chai-1,^38^ and DynamicBind^39^) on a rigorously curated dual benchmark of orthosteric and allosteric ligand-protein complexes obtained from the Major Drug Targets (MDT) dataset,^39^ a rigorously curated set of allosteric ligand complexes from the Kinase Conformation Resource (KinCoRe),^81,82^ a dataset of competitive and allosteric protein kinase inhibitors confirmed by X-ray crystallography,^83^ a high-confidence test set derived from the Allosteric Database (ASD)^66^ using a recently validated rigorous selection protocol,^84^ and validated datasets of protein-ligand orthosteric competitive (PLOC) complexes and protein-ligand allosteric (PLA) complexes.^85^

The benchmarking component systematically evaluates performance on rigorously stratified orthosteric and allosteric datasets, assessing two interconnected tasks that for allosteric ligand binding present a compounded challenge: identification of the ligand-binding pocket and structural prediction of the ligand-binding pose within the pocket. The interpretability layer based on conformational ad mutational frustration analyses provides a quantitative readout of the energetic and evolutionary organization of the ligand binding enabling direct testing of the hypothesis that robustness and accuracy of allosteric ligand predictions can be dictated not only by model architecture and structural memorization of interaction patterns but also by the topological organization of the underlying energy landscape. By linking quantitative predictions of AI models to the landscape-based explainable-AI framework, this study recasts allosteric prediction blind spots not only as algorithmic shortcomings but rather as potential diagnostic indicators of biophysical allosteric grammar.

### Orthosteric binding exhibits unimodal convergence to near-experimental accuracy across diverse architectures

**Figure 2.**
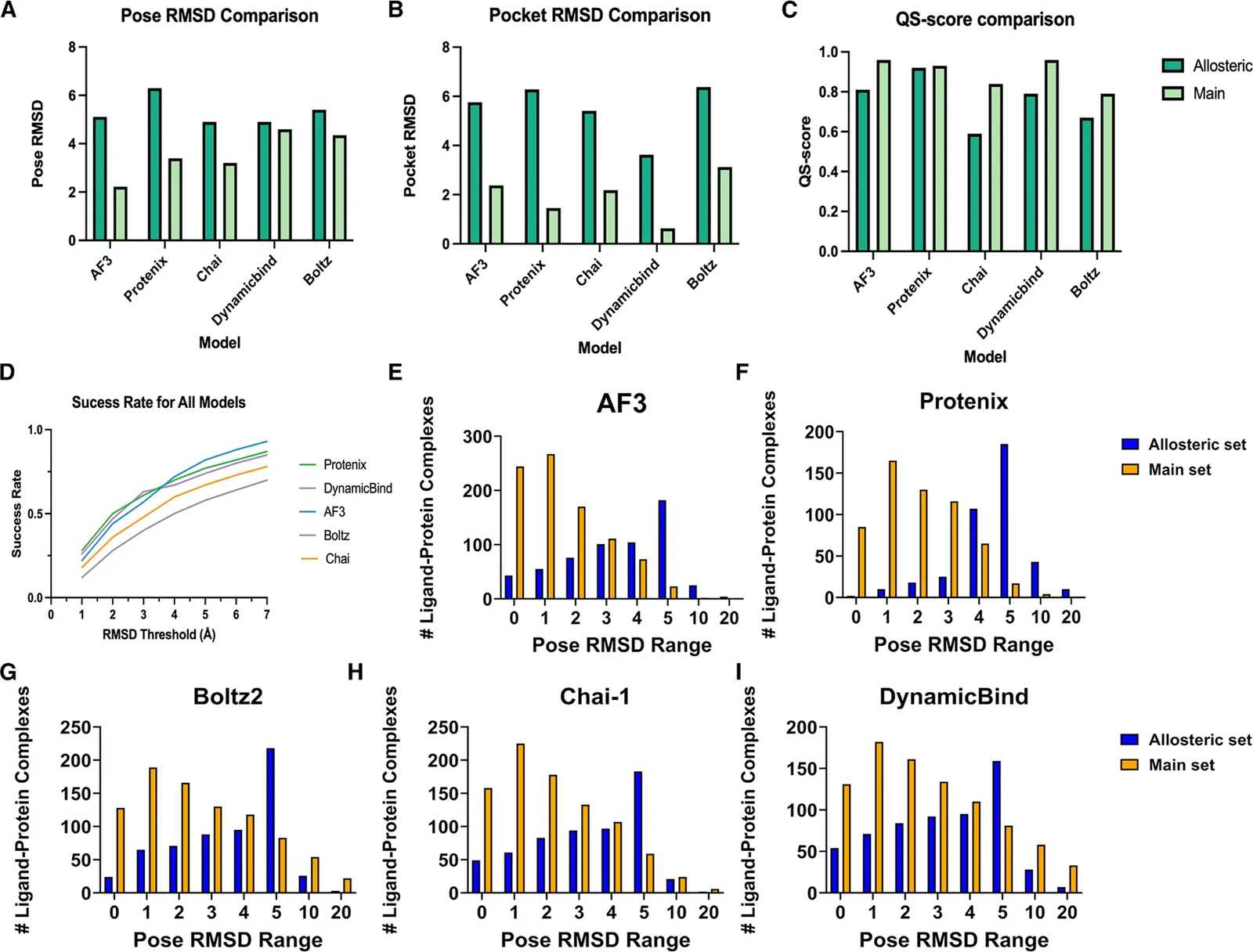
Benchmarking performance of five AI co-folding models on orthosteric versus allosteric ligand binding (A) Mean ligand-pose RMSD (±SD) for orthosteric (light green) and allosteric (dark green) complexes across the five models. Orthosteric errors are low (2.3–4.1 Å), while allosteric errors approximately double (5.2–6.8 Å). (B) Mean pocket RMSD (±SD). Orthosteric pocket RMSD remains ≤3.0 Å; allosteric pocket RMSD rises to 3.5–6.3 Å, indicating difficulty in localizing regulatory pockets. (C) Mean QS score (±SD). Orthosteric complexes achieve near-perfect contact topology recovery (QS > 0.90). Allosteric QS scores remain moderately high (0.70–0.85) despite large geometric errors, revealing decoupling between topology and geometry. (D) Success rate as a function of ligand RMSD threshold. The *x* axis shows the RMSD threshold (Å) used to define a successful prediction. The *y* axis shows the fraction of predictions (oracle selection) with RMSD below that threshold. Each colored line corresponds to one model (AF3, Protenix, Boltz-2, Chai-1, DynamicBind). (E–I) Histograms of ligand-pose RMSD distributions for each model: (E) AF3, (F) Protenix, (G) Boltz-2, (H) Chai-1, and (I) DynamicBind. Orange bars represent orthosteric complexes; blue bars represent allosteric complexes. Orthosteric distributions are sharply peaked at low RMSD (0–2 Å), while allosteric distributions are broad, flat, and shifted to higher RMSD (5–20 Å). The bin width is 2 Å. Note that the *y* axis scales differ between models to best show the shape of each distribution.

We first sought to establish a high-fidelity baseline for canonical orthosteric binding against which allosteric performance could be compared. To this end, we assembled a comprehensive orthosteric reference combining the MDT dataset (412 complexes from kinases, G protein-coupled receptors, nuclear receptors, and ion channels selected for structural challenge requiring pocket RMSD greater than 2.0 Å between apo and holo structures)^39^ together with the PLOC subset adding 1,863 experimentally determined structures with 2,325 binding pockets and 1,647 unique ligands spanning diverse families, all binding competitively to primary, evolutionarily conserved orthosteric sites.^85^ The initial combined orthosteric reference totals 2,275 complexes, capturing the diversity of evolutionarily optimized, geometrically well-defined binding sites. Across all five AI models evaluated on this comprehensive reference, orthosteric ligand-pose predictions converged tightly around native geometries (Figure 2A). AF3, Protenix, and Chai-1 achieved mean ligand-pose RMSD of 2.3–2.8 Å; Boltz-2 and DynamicBind reached mean ligand-pose RMSD of 3.8–4.1 Å (Figure 2A). Pocket RMSD analysis revealed complementary structural fidelity (Figure 2B). DynamicBind achieved the lowest mean pocket RMSD of approximately 1.0 Å, while AF3 and Protenix maintained mean pocket RMSDs below 2.0 Å (Figure 2B). Beyond geometric accuracy, orthosteric predictions achieved near-perfect recovery of native contact topology, with QS scores exceeding 0.90 across all models (Figure 2C) further reinforcing the robust encoding of orthosteric ligand-protein binding pockets and interaction patterns in training data. At the stringent threshold of 2.0 Å for ligand-pose RMSD, both AF3 and Protenix models exceeded 80% success rates, a benchmark reflected in the success rate matrix (Figure 2D), which shows consistently high performance accuracy of orthosteric binding predictions across all models.

The most striking feature emerges from granular distribution analysis (Figures 2E–2G). Across all models, more than 70% of predictions cluster within ligand-pose RMSD ≈ 0–2 Å, forming a sharp unimodal distribution centered on the native pose. This is the signature of a well-defined energetic funnel as the landscape geometry forces sampling trajectories toward a single dominant minimum, enabling different architectural approaches to arrive at the same experimentally validated solution.

### Allosteric binding induces performance degradation across different AI architectures

The same five AI models were evaluated on the assembled allosteric dataset integrating kinase-specific complexes from the KinCoRe database^81,82^ (217 type III/IV allosteric inhibitors), a curated set of high-confidence allosteric kinase inhibitors (136 complexes),^83^ and the PLA dataset containing a total 1,613 structures with 1,830 binding pockets and 1,277 unique ligands spanning 83 Pfam families across >500 organisms.^85^ All these complexes were experimentally validated as authentic allosteric binders with sites spatially distinct from orthosteric pockets. Noteworthy, the 1,830 binding pockets reported in the original PLA subset represent the total number of complexes but not necessarily distinct allosteric sites. To address this, we additionally processed the PLA subset to distinguish total complexes from genuinely distinct allosteric pockets.

First, we analyzed the prediction results for the complete unfiltered set of allosteric complexes obtained from the KinCoRe database and the PLA dataset. The results revealed a notable and systematic degradation in predictive performance. Indeed, the mean ligand-pose RMSD increased to 5.2–6.8 Å across all evaluated AI architectures, effectively doubling the orthosteric error range of 2.3–4.1 Å (Figure 2A). This degradation is not model specific, suggesting that the allosteric blind spot may not only arise from training limitations and algorithmic deficiencies of the examined AI co-folding models but also reflect the unique nature of allosteric binding landscape.

Importantly, the marked underperformance on allosteric binding affects both components of the dual prediction task. Mean pocket RMSD nearly doubled, increasing from 1.4 to 3.0 Å for orthosteric sites to 3.5–6.3 Å for allosteric sites (Figure 2B). Protenix shows the most severe pocket degradation, with mean pocket RMSD rising from 1.5 to 6.2 Å, while even DynamicBind, which is the strongest performer for pocket localization, exhibits a substantial increase from RMSD 1.0 to 3.8 Å (Figure 2B). Our results indicated that AI models are often unable to accurately localize regulatory pockets, and, even when pockets are approximately identified, the predictions of allosteric ligand-binding poses may deviate significantly from experimental solutions. The QS-score analysis further illuminates the observed divergence in predicting orthosteric and allosteric ligand-binding poses (Figure 2C). Indeed, while orthosteric complexes achieve near-perfect contact recovery with QS scores exceeding 0.90, allosteric QS scores remain moderately high at 0.70–0.85 despite geometric error, revealing that AI models may identify the correct interaction partners but are often unable to accurately resolve their spatial arrangement into a unique binding geometry. This indicates that, even when the proper interaction partners are recognized for allosteric ligands, the energetic guidance is often insufficient to enforce geometric convergence.

Success rates for allosteric ligand binding at the stringent 2.0-Å ligand-pose RMSD threshold collapse to 25%–35% across AI models (Figure 2D), and the tightly unimodal orthosteric distributions transform into broad, flat profiles extending toward RMSD > 10 Å (Figures 2E–2G). AF3 predictions skew strongly toward the RMSD 5- to 10-Å bin with a pronounced long tail beyond 20 Å. Chai-1 displays a modest peak at RMSD = 2–3 Å followed by rapid decay, consistent with the inability of sequence-based priors to compensate for missing coevolutionary signals at evolutionarily permissive allosteric sites. Boltz-2 exhibits the most severe degradation, with predictions concentrated in the RMSD = 5- to 10-Å range. DynamicBind retains a small peak at RMSD = 2–3 Å for allosteric ligands, reflecting its emphasis on induced-fit modeling, yet still underperforms for the majority of complexes. Model-specific patterns in pose degradation are also evident (Figure 2A). AF3 and Protenix, the top performers on orthosteric sites, exhibit the largest absolute degradation, with mean ligand-pose RMSD increasing by approximately 4.1 Å (Figure 2A). DynamicBind shows comparative resilience with mean ligand-pose RMSD ~4.5 Å yet remains far from experimental accuracy, underscoring that explicit flexibility modeling may be insufficient to overcome intrinsic biophysical constraints underlying the nature of allosteric binding. Intriguingly, it is tempting to suggest the architectural features of AI models optimized for extracting maximal signal from high-quality orthosteric input could become liabilities when confronted with energetic degeneracy of allosteric binding.

**Figure 3.**
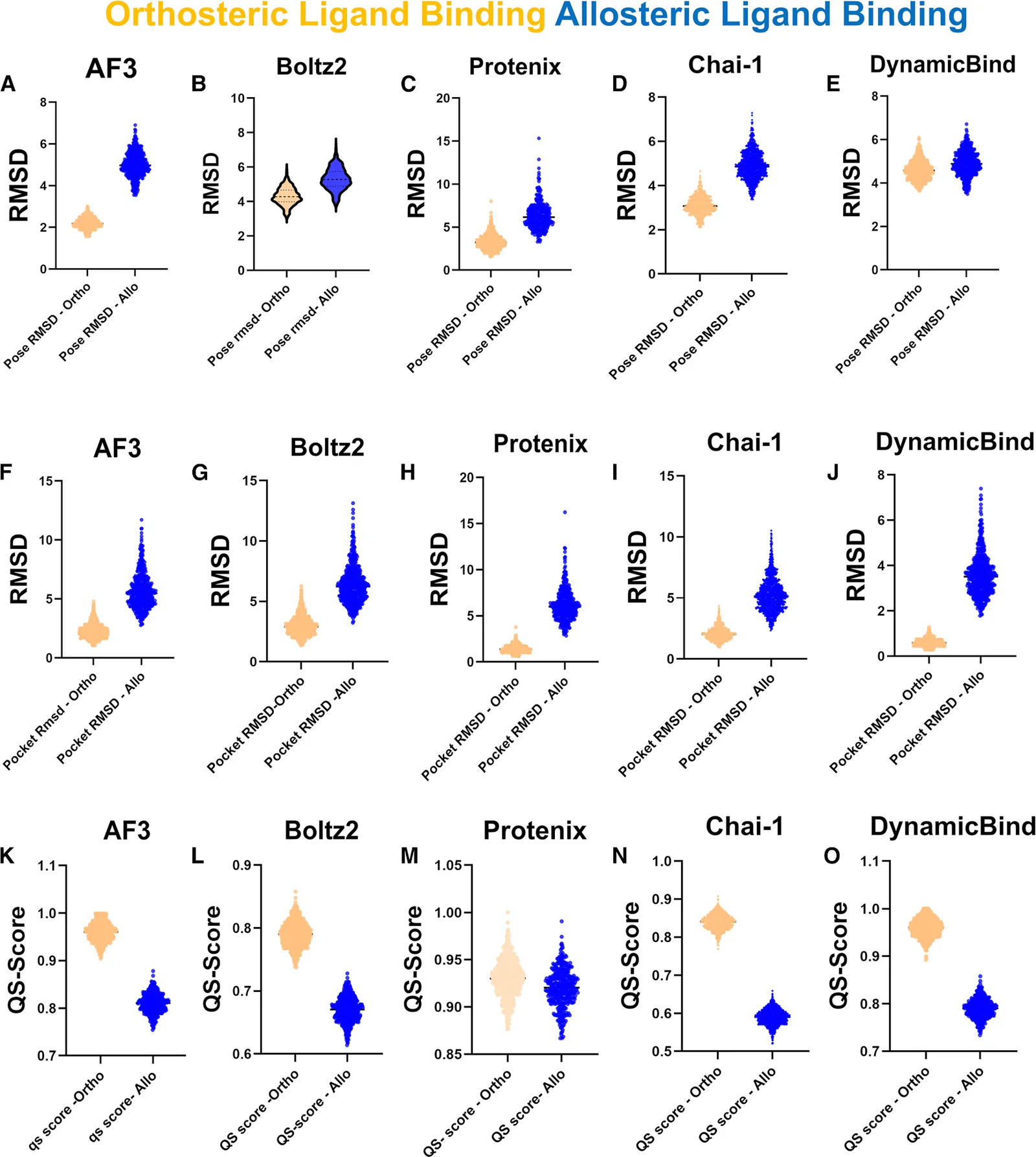
Distributional comparison of AI model performance for orthosteric versus allosteric ligand binding across pose accuracy, pocket localization, and contact topology Violin plots summarize prediction performance for five AI co-folding models (AF3, Protenix, Boltz-2, Chai-1, and DynamicBind), contrasting orthosteric complexes (orange) with allosteric complexes (blue). (A–E) Ligand-pose RMSD distributions. For all five models, orthosteric predictions (orange) exhibit narrow, symmetric distributions centered below RMSD ~3.0 Å, indicating high geometric precision and unimodal convergence toward the native pose. In contrast, allosteric predictions (blue) display broad, flattened distributions extending beyond RMSD = 10 Å, reflecting systematic loss of geometric accuracy and the emergence of multiple distinct, non-native solutions. AF3 and Protenix show the strongest broadening for allosteric sites, whereas DynamicBind exhibits the most compressed allosteric distribution, consistent with its partial resilience to induced-fit effects. (F–J) Pocket RMSD distributions. Orthosteric binding pockets (orange) are localized with higher precision, yielding tight distributions typically below ~3.0 Å across models. Allosteric pockets (blue) show markedly wider and right-shifted distributions, confirming difficulty in correctly identifying diverse and weakly conserved allosteric binding sites. (K–O) QS score (contact topology) distributions. Orthosteric complexes (orange) achieve near-perfect recovery of native contact networks (QS > 0.90) for all models, indicating faithful reconstruction of interaction topology. Allosteric complexes (blue) retain only moderate QS scores (0.70–0.85) revealing a systematic decoupling between contact topology recovery and geometric precision.

While the statistical analysis revealed the divergence and quantified magnitude of the prediction performance gap (Figure 2), the contrast of the underlying distributional shapes between orthosteric and allosteric ligand-binding predictions provided further insight into mechanistic origin of this dichotomy (Figure 3). The narrow, symmetric, unimodal distributions for orthosteric predictions (Figures 3A–3E) reflect convergence to a unique energetic minimum, while the broad, flat, frequently bimodal distributions for allosteric predictions reflect the rugged, degenerate landscape that permits multiple distinct yet energetically comparable binding modes. For all five AI models, orthosteric ligand-pose RMSD (Figures 3A–3E), pocket RMSD (Figures 3F–3J), and QS-score distributions (Figures 3K–3O) exhibit narrow, symmetric distributions centered below RMSD ≈ 3.0 Å, indicating high prediction precision and unimodal convergence toward the native pose. Narrow interquartile ranges and the absence of extended tails indicate robust geometric convergence and demonstrate that orthosteric binding constitutes a well-posed prediction problem for AI models. In contrast, allosteric predictions display broad, flattened distributions extending beyond RMSD = 10 Å, reflecting loss of geometric accuracy and the emergence of multiple distinct, non-native solutions. AF3 and Protenix show the strongest broadening for allosteric sites, whereas DynamicBind exhibits the most compressed allosteric distribution (Figure 3).

The divergence is particularly pronounced for ligand-pose RMSD (Figures 3A–3E). AF3 and Protenix show the most dramatic transformation: their sharply peaked orthosteric violins (median ligand RMSD ≈ 2.5 Å, interquartile range <1.5 Å) broaden into highly diffuse allosteric distributions spanning approximately 2–20 Å, with medians exceeding 6 Å. For pocket RMSD (Figures 3F–3J), the degradation is even more uniform: all five models exhibit median allosteric pocket RMSD values above 6 Å, reflecting the universal difficulty in localizing diverse allosteric binding sites. For QS score (Figures 3K–3O), orthosteric predictions achieve near-perfect recovery (QS > 0.90), while allosteric QS scores remain moderately high (0.70–0.85) despite significant geometric errors, revealing a systematic decoupling between topology and geometry. While some allosteric pockets are localized with near-orthosteric precision, others are completely missed. This heterogeneity could reflect the intrinsic diversity of allosteric sites and lack of consistent geometric or evolutionary signature that can be assigned to allosteric binding.

Hence, the results demonstrate that prediction accuracy for orthosteric and allosteric complexes not only differs in magnitude and statistical structure but could also reflect mechanistic differences linked to the physical nature of allosteric binding landscapes (Figure 3). Orthosteric predictions form tight, symmetric distributions with narrow interquartile ranges and short tails across all three metrics—ligand-pose RMSD, pocket RMSD, and QS score—indicating that binding geometry and interaction topology are simultaneously well constrained. In contrast, allosteric distributions are broad, flat, and frequently bimodal, with long tails extending to high RMSD values while QS scores remain comparatively elevated. This pattern suggests that allosteric prediction degradation is not simply a matter of increased noise or random error but can reflect a dichotomy between interaction recovery and geometric convergence. Together, these results suggest that the observed performance gap of AI models on allosteric ligand binding could result from a compounded challenge of simultaneous mislocalization of binding pockets and lack of geometric convergence. A persistent separation between orthosteric and allosteric distributions suggests that an “allosteric blind spot” can reflect a complex balance of multiple factors including training biases toward statistically dominant orthosteric binding, the effects of structural memorization of conserved patterns presented by canonical binding, as well as peculiarities of the allosteric binding landscapes lacking a unified structural or evolutionary signature.

### Orthosteric-allosteric binding mode prediction divergence remains under oracle, top-ranked, and average selection metrics

The observed performance gap observed in the original allosteric benchmark could, in principle, be influenced by dataset-specific biases (e.g., over-representation of certain protein families, pocket redundancy, or temporal data leakage). To rule out these possibilities and to evaluate the generalizability of our findings, we evaluated all five AI co-folding models on two additional, independently curated allosteric datasets. We employed a high-confidence test set derived from ASD dataset.^66^ Using a recently validated selection protocol,^84^ from the list of 3,102 proteins, we selected human proteins with a “modulator_class” annotation to focus on small-molecule allosteric binding sites, resulting in a total of 206 proteins. Quality control and allosteric binding-site rigorous mapping reduced the set to 141 proteins spanning 182 unique sites. Second, we explored a non-redundant set of distinct allosteric pockets extracted from the PLA subset after clustering by UniProt ID, pocket residue composition, and geometric similarity (Cα RMSD ≤2.0 Å) resulting in 182 unique allosteric pockets spanning 115 distinct UniProt entries. For each unique pocket, we selected the highest-resolution representative structure.

All results reported thus far focused primarily on “oracle” selection, where, for each complex, we chose the single prediction with the lowest ligand RMSD among multiple independent runs. This approach establishes an upper bound on model capability. To assess realistic performance and to test whether the orthosteric-allosteric gap persists under less optimistic selection, we evaluated two additional strategies: (1) model-selected top-ranked—the single prediction with the highest native confidence score (ipTM for AF3, Protenix, Boltz-2, and Chai-1; composite for DynamicBind); and (2) average—the mean RMSD over all 25 predictions, which provides a conservative lower bound.

Strikingly, for orthosteric sites, the difference between oracle and average performance is minimal across all models and datasets. For example, on the original benchmark, AF3’s oracle RMSD is 2.3 Å, while its average RMSD is 3.1 Å—a difference of only 0.8 Å. Success rates at 2 Å drop from 82% (oracle) to 58% (average), but the key point is that even the average orthosteric prediction is highly accurate (RMSD <3.5 Å for all models except Boltz-2) (Table S1). This consistency reflects the fact that orthosteric binding landscapes are dominated by a single deep energetic funnel; all sampled predictions converge near the native pose, making the choice of selection strategy largely irrelevant.

For allosteric sites, the oracle-average gap is larger (typically 1.5–2.5 Å), but the fundamental conclusion remains unchanged. On the original benchmark, for AF3 predictions, oracle allosteric RMSD is 6.1 Å, while its average allosteric RMSD is 7.5 Å. Success rates at 2 Å drop from 29% (oracle) to 14% (average). Critically, even the average allosteric RMSD (>7 Å) is more than double the average orthosteric RMSD (<3.5 Å), and the success rate remains near or below 20% for all models (Table S1). The decoupling between QS score and RMSD persists, and allosteric QS scores remain moderately high (0.65–0.70) even for average predictions, while geometric error remains catastrophic.

The same analysis repeated on the ASD and PLA datasets (Table S1) yields identical conclusions. The orthosteric-allosteric gap is highly significant (*p* < 0.001) regardless of selection method. The small oracle-average gap for orthosteric sites across all datasets (typically <1.0 Å) demonstrates that models are not merely “lucky” in their best predictions—they are consistently accurate. For allosteric sites, the larger gap reflects the intrinsic flatness of the energy landscape, where multiple distinct poses are sampled; even the best predictions remain inaccurate, and the average is worse.

Confidence scores correlate strongly with RMSD for orthosteric sites but weakly for allosteric sites. Spearman correlation coefficients (ρ) between predicted confidence and actual RMSD are ρ ≈ −0.5 to −0.6 for orthosteric complexes across all models, indicating that models can reliably rank their own predictions. For allosteric complexes, ρ drops to −0.2 to −0.3, consistent with the flat, degenerate landscape where multiple poses have similar confidence but widely varying accuracy. This further reinforces that the allosteric blind spot is not a ranking problem but a fundamental limitation of the underlying energy landscape.

This analysis shows that the oracle results do not overestimate the gap but rather simply represent the upper bound of prediction accuracy. Even under the most conservative selection (average of all predictions), orthosteric sites are predicted with high accuracy while prediction success of allosteric binding modes remains systematically reduced. The performance dichotomy is robust to selection strategy, dataset, and model architecture.

### Ligand-binding prediction dichotomy and allosteric blind spot persist across ASD-derived and non-redundant PLA allosteric datasets

Here, we provide a detailed distributional analysis for the ASD and PLA datasets using the two extreme selection strategies (oracle and average). While the previous chapter explores question “does the gap survive different selection methods?” in this section we examine “how does the gap look in independent allosteric datasets when we examine the full distributions?” To provide a more detailed view of prediction behavior on independent ASD and PLA allosteric datasets, we evaluated all five AI co-folding models in detail by focusing on the two extreme selection strategies—oracle (establishing an upper bound on model capability) and average (providing a conservative lower bound). The violin distribution plots for the oracle strategy are shown in Figure S1, while those for the average strategy are shown in Figure S2. The distribution plots of ligand-pose RMSDs for each model on the ASD dataset and the non-redundant PLA set under the oracle selection (Figures S1A–S1E) are similar to those obtained in the original allosteric benchmark (Figures 3A–3E), displaying broad, right-skewed profiles with medians between 5 and 7 Å and long tails extending beyond 10 Å (Tables S2 and S3). Success rates at the 2-Å threshold remain below 35%. AF3 achieves 28% on ASD and 30% on PLA; Protenix 26% on ASD and 27% on PLA; Boltz-2 23% on ASD and 24% on PLA; Chai-1 31% on ASD and 32% on PLA; DynamicBind 33% on ASD and 34% on PLA (Figures S1A–S1E; Tables S2 and S3). These oracle success rates are marginally higher than those reported for the original allosteric benchmark, confirming that even the best possible selection is unable to close the allosteric accuracy gap.

We recomputed the within-pocket variance analysis using the ASD and non-redundant PLA datasets under the oracle strategy. For each model, we identified allosteric pockets with at least three distinct ligand complexes (based on UniProt ID and pocket residue composition). For each such pocket, we calculated the standard deviation of ligand-pose RMSD across the available ligand instances. The mean within-pocket SD was then averaged over all qualifying pockets (≥3 ligands) (Table S4). Notably, within-pocket variance for pockets with multiple ligand instances is ≤0.35 Å for all models (Table S4), confirming that the choice of representative ligand does not affect the conclusions. Moreover, the oracle RMSD distributions from ASD, PLA, and the original allosteric benchmark are statistically indistinguishable (Kolmogorov-Smirnov test, *p* > 0.3 for all pairwise comparisons). Mirroring the ligand-pose results, pocket RMSD distributions under oracle selection are broad and right-skewed, with medians ranging from 3.8 Å (DynamicBind) to 5.8 Å (Boltz-2) on ASD, and from 3.7 Å (DynamicBind) to 5.6 Å (Boltz-2) on the non-redundant PLA set (Figures S1F–S1J). These values are substantially higher than orthosteric pocket RMSD medians (<2.0 Å; Figure 2B) and are statistically indistinguishable from the original allosteric benchmark (Kolmogorov-Smirnov test, *p* > 0.3 for all pairwise comparisons).

Critically, the decoupling between contact topology and geometric precision, which is a signature of allosteric binding, persists under oracle selection on both independent allosteric datasets. Figures S1K–S1O shows violin distributions of QS scores for all five models. Despite the large geometric errors (median ligand RMSD >5 Å), QS scores remain moderately high, ranging from 0.61 to 0.74 on ASD and from 0.62 to 0.75 on the non-redundant PLA set (Tables S2 and S3). For example, AF3 achieves a median QS score of 0.68 on ASD and 0.69 on PLA, while DynamicBind reaches 0.74 on ASD and 0.75 on PLA—values comparable to those observed in the original allosteric benchmark (0.70–0.85). This systematic preservation of native contact topology, even when the predicted ligand pose is geometrically inaccurate, confirms that AI models recover the vocabulary of allosteric interactions (which residues should contact the ligand) but fail to resolve the syntax (the precise three-dimensional arrangement). The oracle QS-score distributions on ASD and PLA are statistically indistinguishable from the original benchmark (*p* > 0.3), further reinforcing the robustness of the decoupling phenomenon.

To determine whether the allosteric blind spot remains under the most conservative selection strategy, we repeated the entire analysis using the average RMSD over all 25 predictions per complex. The violin distribution plots for the average strategy are shown in Figures S2A–S2E. As may be expected, the average ligand RMSD distributions are shifted upward relative to the oracle distributions but remain generally similar with the qualitative dichotomy largely unchanged. For all five models, the average allosteric RMSD medians on ASD and PLA range between 6.5 and 8.5 Å—more than double the average orthosteric RMSD values (<3.5 Å from the original benchmark). Success rates at 2 Å drop further: on ASD, AF3 achieves 13%, Protenix 11%, Boltz-2 9%, Chai-1 16%, and DynamicBind 17%; on PLA, the corresponding rates are 15%, 13%, 11%, 17%, and 18% (Tables S2 and S3; Figures S2A–S2E). Notably, even the best-performing model under average selection (DynamicBind, with 17%–18% success at 2 Å) fails to reach 20%, and its median RMSD remains above 5.5 Å. The within-pocket variance analysis under average selection (Table S4) yields the same conclusion: mean within-pocket SD is ≤0.40 Å for all models, confirming that the choice of representative ligand does not bias the results. The average RMSD distributions from ASD and PLA are statistically indistinguishable from each other and from the original allosteric benchmark’s average distributions (Kolmogorov-Smirnov test, *p* > 0.3 for all pairwise comparisons). Pocket RMSD under average selection (Figures S2F–S2J) shows medians between 4.2 Å (DynamicBind) and 6.2 Å (Boltz-2) on ASD, and between 4.0 Å and 6.0 Å on PLA—again substantially higher than orthosteric pocket RMSD medians and similar to the original allosteric benchmark averages.

Most importantly, the QS score vs. RMSD decoupling is even more striking under average selection (Figures S2K–S2O). Despite the larger geometric errors (median ligand RMSD now >7 Å for several models), QS scores remain only slightly reduced relative to the oracle case. On ASD, average QS scores range from 0.58 (Boltz-2) to 0.71 (DynamicBind); on PLA, from 0.59 to 0.72. For AF3, the average QS score on ASD is 0.64, only 0.04 lower than its oracle QS score of 0.68, while the average ligand RMSD (7.3 Å) is 1.2 Å higher than the oracle RMSD (6.1 Å). This persistent decoupling confirms that, even when the model is forced to average over all its predictions, it still recovers native-like contact topologies while often failing to produce accurate ligand poses.

In summary, the orthosteric-allosteric performance dichotomy is robust to both dataset choice (ASD, PLA, original benchmark) and prediction selection strategy (oracle vs. average). Oracle analysis establishes the upper bound: even the best possible prediction per complex fails to achieve consistently accurate allosteric poses (median RMSD >5 Å, success rate <35%). Average analysis provides the conservative lower bound: when all predictions are considered, allosteric accuracy collapses further (median RMSD >6.5 Å, success rate <20%), yet QS scores remain moderately high. The allosteric blind spot is therefore not a consequence of dataset bias or biased evaluation selection but could rather reflect a general, architecture-independent limitation of current AI co-folding models.

### Allosteric binding prediction improves with structural coverage but remains below orthosteric levels

A potential concern is that the observed performance gap could be inflated by inadvertent train-test leakage—for example, if allosteric complexes in the benchmark share high sequence identity with structures used to train the AI models, or if they were deposited before the training data cutoff. To address this, we applied the same rigorous temporal (post-2020) and sequence similarity (≤30% identity) filters that were used for the orthosteric benchmark to the allosteric dataset. Filtering for temporal and sequence similarity confirms no data leakage. After this filtering, the orthosteric reference comprised 2,184 high-confidence complexes (slightly reduced from the original 2,275). For the allosteric dataset, the same filters were applied to the kinase-specific allosteric complexes (from KinCoRe and the curated kinase repository), yielding 321 high-confidence kinase allosteric complexes. Separately, the non-redundant PLA set (182 distinct allosteric pockets) was also filtered using the same criteria, resulting in no further reduction because the representative structures were already post-2020 and had low sequence similarity. Thus, the combined filtered allosteric dataset for this analysis comprised 503 complexes. The ASD-derived test set was not included in this filtered allosteric benchmark because it was curated as an independent cross-validation set following a distinct protocol rather than as a component of the main filtered dataset.

As shown in Table S5, performance metrics on this filtered allosteric set remained virtually unchanged from the original analysis: mean ligand-pose RMSD ranged from 5.3 Å (DynamicBind) to 6.8 Å (Boltz-2), success rates at 2 Å were between 24% and 33%, and QS scores were 0.63–0.76. Paired *t* tests comparing the original and filtered results showed no statistically significant differences for any model or metric (*p* > 0.3 for all comparisons). This additional analysis demonstrates that the allosteric blind spot is not a consequence of train-test leakage or temporal overfitting; the performance gap is present even when restricting to structures deposited after the training data cutoff and to sequences with low similarity to training examples.

We have also examined whether model performance may correlate with structural coverage (number of deposited structures for a given allosteric site). This question was evaluated using the non-redundant allosteric PLA pocket set (182 pockets). For each pocket, we queried the PDB for the number of distinct ligand-bound structures sharing the same UniProt ID and pocket residue composition. The 182 distinct allosteric pockets were stratified by PDB instance frequency. Pockets were classified as low-coverage (≤5 PDB entries, *n* = 78), medium-coverage (6–20, *n* = 62), high-coverage (21–40, *n* = 32), or ultra-high-coverage (>40, *n* = 10). Table S6 reports the oracle (best-of-25) ligand-pose RMSD and success rate at 2 Å for all five models within each stratum (the same selection used in the main benchmark). A trend toward better performance on higher-coverage allosteric sites is observed across all models. For the ultra-high-coverage stratum (e.g., HSP90, CDK2), AF3 achieves a mean RMSD of 4.2 Å and 45% success, Chai-1 reaches 4.0 Å and 48% success, and DynamicBind attains 3.5 Å and 52% success—substantially better than low-coverage allosteric sites (Table S6). However, even in the high-coverage stratum featuring these most heavily sampled allosteric sites, performance remains below orthosteric levels. For AF3, orthosteric RMSD is 2.3–2.8 Å with >80% success; the best allosteric ultra-high-coverage result (DynamicBind: 3.5 Å, 52% success) still falls short. Moreover, the success rate at 2 Å for ultra-high-coverage pockets (45%–52%) is less than two-thirds of orthosteric success (>80%). Critically, even for allosteric sites that are extremely well represented in the PDB, such as HSP90 with >50 deposited allosteric ligand complexes, AI binding models consistently underperform in achieving sufficient accuracy (RMSD < 2 Å) and high success rates across all examined approaches. Importantly, however, we observed that allosteric prediction accuracy does improve with the increasing structural coverage. For low-coverage pockets, the mean RMSD exceeds 6 Å and success rates at 2 Å are only 20%–30%, while, for high-coverage pockets (>40 entries), RMSD drops to 3.5–4.5 Å and success rate rises to 45%–52%. This systematic improvement strongly suggests that structural memorization contributes measurably to allosteric binding prediction for a well-studied system. Models may learn recurrent pocket geometries and ligand-binding modes for allosteric sites that are heavily represented in the training data (e.g., HSP90, CDK2). These results are in strong alignment with recent studies that illuminated a role for structural memorization.^86,87^ One of these studies evaluated several AI co-folding methods using newly introduced benchmark dataset that comprises 2,600 high-resolution protein-ligand systems showing that current AI approaches largely memorize ligand poses from their training data.^86^ Our findings that allosteric prediction accuracy may improve with increasing structural coverage are also consistent with recent work that demonstrated that AF2 predictions of fold-switched conformations are driven by memorization of training examples rather than physical reasoning.^87^

Moreover, this illuminating study showed that structural memorization has significantly greater effect on predictions than information inferred from coevolutionary restraints, further stressing the need for incorporation of physics-based priors into AI models. Our data confirm that structural memorization contributes measurably to allosteric binding prediction, especially for well-studied ligand-protein systems with significant presence in structural databases. Importantly, however, we also found memorization effect alone cannot explain the persistent performance gap between orthosteric and allosteric ligand-binding predictions. Even for the most heavily sampled allosteric sites (>50 PDB entries), models are unable to reach orthosteric-level accuracy (RMSD > 3.5 Å, success < 55%). In contrast, orthosteric sites with far fewer training examples routinely achieve high-accuracy predictions with RMSD < 1.5–2 Å and >80% success. This dissociation indicates that the limiting factor is not simply the number of examples but rather the intrinsic biophysical organization of the binding site. The persistent underperformance even for ultra-high-coverage allosteric sites points to an intrinsic biophysical limit suggesting that the underlying energetic organization of allosteric binding sites may impose some fundamental constraints that cannot be overcome by simply providing more training examples.

To ensure that the observed performance gap is not an artifact of differences in protein-family composition, ligand properties, pocket burial, or conformational heterogeneity, we performed several matched and stratified analyses. The most straightforward way to control for protein-family composition is to compare orthosteric (ATP-binding) and allosteric (type III/IV) pockets within the same protein family—the human kinome. For orthosteric comparisons, we used type I/II ATP-competitive inhibitors from the same kinases. Within this family-matched set, the performance gap remained large and highly significant: orthosteric ligand RMSD (AF3) was 2.4 ± 0.3 Å (79% success at 2 Å), while allosteric RMSD was 5.9 ± 1.5 Å (26% success; *p* < 0.001) (Table S7). Hence, even when restricting to the same protein kinase family, the performance gap remains highly significant, ruling out the possibility that the gap is driven by comparing different protein families.

We also matched binding pockets by depth and solvent accessibility. Allosteric pockets might be more surface exposed than orthosteric pockets. We used Fpocket^46^ to compute for each pocket the distance from pocket center to protein surface and relative solvent accessibility of pocket residues. The created matched subsets include deep buried pockets (distance > 5 Å, accessibility < 10%) and surface-exposed pockets (distance < 3 Å, accessibility > 30%). Even when restricting to deeply buried pockets orthosteric predictions remained accurate (RMSD ≈ 2.5 Å) while allosteric predictions remained relatively poor with RMSD ≈ 5.8 Å (Table S8).

Allosteric ligands may differ from orthosteric ligands in molecular weight (MW), logP, number of rotatable bonds, or hydrogen-bond (H-bond) donors/acceptors. We performed propensity-score matching using nearest-neighbor matching on five properties: MW, logP, rotatable bonds, H-bond donors, and H-bond acceptors. After matching, we obtained 502 orthosteric-allosteric pairs with statistically indistinguishable ligand property distributions (all *p* > 0.3). In the matched set, orthosteric ligand RMSD remained 2.5 ± 0.5 Å (success 78%), while allosteric RMSD remained 6.1 ± 1.7 Å (success 27%), *p* < 0.001 (Table S9). Thus, ligand properties are unlikely to have an appreciable effect on the prediction performance gap.

Allosteric ligand binding might involve larger backbone rearrangements than orthosteric binding, making prediction harder. We retrieved apo structures (or closest homologs) for a representative subset and computed global Cα RMSD between apo and holo states. We stratified into three bins: small (RMSD < 1.0 Å), medium (1.0–2.0 Å), large (>2.0 Å). Noteworthy, the success rate for allosteric ligand-binding poses showed moderate increase in the success rate for complexes with reduced protein conformational changes. However, this improvement is marginal, and, even when restricting to complexes with minimal backbone rearrangement (<1.0 Å), the gap persists (orthosteric 2.2 Å vs. allosteric 5.4 Å) (Table S10).

Finally, the original orthosteric dataset (2,275 complexes) is larger than the original unfiltered allosteric dataset (1,966 complexes). To ensure that the gap is not due to unequal statistical power or diversity, we randomly subsampled the orthosteric dataset to match the allosteric dataset size and repeated the performance comparison. We performed 1,000 bootstrap subsamples. In all 1,000 subsamples, the mean orthosteric ligand RMSD remained below 3.0 Å (range 2.5–2.9 Å), and the mean allosteric ligand RMSD remained above 5.5 Å (range 5.4–6.2 Å). The gap was always highly significant (*p* < 0.001) (Table S11). These independent matched or stratified analyses, each controlling for a different potential confounder (protein family, pocket burial, ligand properties, conformational change magnitude, and dataset size), indicated that the orthosteric-allosteric performance gap remained large, significant, and directionally unchanged to compositional differences between the datasets.

To summarize, the convergence of the pose and pocket RMSD distributions across three independent allosteric datasets suggests that the allosteric blind spot is not an artifact of dataset composition, temporal filtering, pocket overcounting, or ligand selection. Instead, it reflects among other reasons the intrinsic property of allosteric binding landscapes lacking well-defined directional energetic funnels for pose convergence and distinctive geometric signatures for pocket localization. These findings support the conclusion that the predictive difficulties faced by current AI models are not only linked to structural memorization and training limitations effects but may also be associated with the biophysical landscape organization of allosteric ligand binding.

### Contact topology recovery decouples from geometric precision in allosteric complexes

Having established that the allosteric blind spot including the persistent decoupling between geometry and topology holds across independent benchmarks, we now examine this decoupling in detail using the original allosteric dataset. To disentangle geometric failure from interaction failure, we quantified recovery of native contact topology using QS scores, which measure agreement between predicted and experimental interaction networks independently of absolute pose geometry. We examine whether AI models can recover correct contact topology even when geometric accuracy collapses by analyzing QS scores independently of pose RMSD. For orthosteric complexes, QS scores exceed 0.90 across all five models, with AF3 and Protenix approaching 0.95 (Figure 2C). This near-perfect recovery demonstrates that canonical orthosteric interaction patterns are robustly encoded and tightly coupled to geometric accuracy: when models predict correct contacts, they also converge to the native pose. In sharp contrast, allosteric complexes exhibit a striking decoupling between topology and geometry. Despite ligand-pose RMSD frequently exceeding 5 Å, QS scores remain moderately high (0.70–0.85) for AF3, Protenix, and DynamicBind (Figure 2C). Allosteric QS-score distributions show only a modest downward shift relative to orthosteric values and remain relatively tight, even as geometric error becomes extreme. This indicates that models frequently identify the correct regulatory region and reconstruct qualitatively native interaction networks but fail to resolve these interactions into a unique geometric arrangement.

This decoupling is visualized systematically in the bulb scatterplots (Figures S3 and S4). Note that the axes are scaled differently between the two figures: orthosteric plots (Figure S3) use a narrow RMSD range (0–6 Å) because predictions are highly accurate, whereas allosteric plots (Figure S4) extend to >20 Å to capture the full spread of errors. For orthosteric complexes (Figure S3), predictions cluster densely in the low-RMSD (<3 Å), high-QS (>0.9) quadrant, reflecting strong coupling between interaction topology and geometry. For allosteric complexes (Figure S4), the distribution spreads dramatically: ligand and pocket RMSDs span a wide range of 4–10 Å, while QS scores remain elevated (0.7–0.9). At first glance, the bulb scatterplots of QS score versus RMSD (Figures S1 and S2) may appear similar, with considerable overlap between orthosteric and allosteric predictions. However, the underlying distributions are statistically distinct. Orthosteric points cluster densely in the low-RMSD (<3 Å), high-QS (>0.9) quadrant (Figure S3), while allosteric points spread horizontally to RMSD values exceeding 15 Å while maintaining QS scores of 0.70–0.85 (Figure S4). The orthosteric RMSD is sharply unimodal near 1–2 Å, whereas allosteric RMSD is broad and flat. Furthermore, the Spearman correlation between QS score and RMSD drops from ρ ≈ −0.65 in orthosteric complexes to ρ ≈ −0.25 in allosteric complexes, quantitatively confirming the decoupling. Thus, both types of predictions can achieve moderate QS scores; the critical difference lies in whether a unique low-RMSD geometry is enforced. For allosteric complexes, this produces a characteristic horizontal decoupling for allosteric complexes where points with moderate to high QS scores (0.70–0.85) span a wide range of RMSD values (from ~2 Å to >15 Å), confirming that models recover the correct interaction partners (high QS) but fail to resolve a unique geometry (high RMSD).

This pattern is conserved across all five architectures, confirming that the decoupling is not model specific but reflects an intrinsic property of allosteric binding. This behavior provides the central mechanistic insight of the benchmark. Allosteric binding prediction fails not because models cannot identify relevant interaction partners but because they cannot resolve the energetic specificity required for geometric convergence. Evolutionary pressures acting on allosteric sites prioritize regulatory flexibility and ensemble redistribution rather than tight energetic optimization, yielding landscapes in which multiple geometrically distinct conformations satisfy similar interaction topologies. Based on these findings, we argue that AI models can recover the vocabulary of allosteric interactions (which residues contact the ligand) but cannot determine the syntax (their precise spatial arrangement), because several syntactically distinct solutions are energetically comparable. This interpretation is exemplified by representative structures in Figure S5. For orthosteric complexes, predicted poses align closely with experimental conformations across architectures, typically within RMSD = 1–2 Å (Figure S5). For allosteric complexes, predictions diverge substantially, adopting alternative ligand orientations or misplacing the pocket entirely, even when native contacts are partially preserved.

Granular analysis further reveals that a substantial fraction of orthosteric predictions achieve high accuracy regime with RMSD < 1.0 Å, approaching crystallographic uncertainty (Figure S6). AF3 achieves the highest proportion of ultra-precise predictions, with 72% of orthosteric complexes below 2.0-Å RMSD and a large fraction below 1.0 Å, reflecting its ability to exploit steep energetic funnels. Chai-1 shows comparable enrichment in the high-precision regime, consistent with its reliance on evolutionary priors that reinforce canonical geometries. Boltz-2 exhibits fewer predictions with RMSD below 1.0 Å from the crystallographic position, reflecting its emphasis on confidence calibration rather than maximal geometric refinement (Figure S6). This hierarchy—in which AF3 and Chai-1 lead in the ultra-high-precision regime, followed by Boltz-2—mirrors the architectural tradeoffs observed throughout this study. Models that excel at extracting maximal signal from steep funnels achieve exceptional accuracy on orthosteric sites, but this very sensitivity may become a liability when confronted with the promiscuous landscapes of allosteric binding. QS-score analysis reveals a fundamental dichotomy.

Taken together, the benchmarking results reveal a striking and reproducible dichotomy across metrics, datasets, and architectures. For orthosteric sites, the evidence is unequivocal: unimodal RMSD distributions centered at 1–2 Å, tight coupling between pocket localization and pose accuracy, near-complete recovery of contact topology (QS > 0.9), and uniform performance across all five models, which are hallmarks of binding governed by steep energetic funnels and frustration quenching. For allosteric sites, an equally consistent but opposite regime emerges. Mean ligand-pose RMSD doubles to 5–7 Å, success rates at the 2.0-Å threshold collapse to 25%–35%, and narrow unimodal distributions broaden into flat or multimodal profiles extending beyond 20 Å. Pocket RMSD also increases, and, although contact topology recovery remains moderately high (QS = 0.70–0.85), this stands in sharp contrast to the accompanying geometric failure. Despite different inductive biases across models, the consistent nature of the observed prediction gaps indicates that allosteric blind spot is unlikely to be solely algorithmic in origin. Instead, it points to intrinsic biophysical differences in the structural and evolutionary organization of orthosteric and allosteric sites.

### Energy-landscape architecture encodes the physical basis of explainable AI: Ligand-induced frustration quenching creates energetic funnels for orthosteric ligand binding

By overlaying AI prediction outcomes with frustration-based landscape profiles, we link model performance on orthosteric and allosteric ligand binding to the physical organization of their underlying energy landscapes, connecting benchmark evaluations with mechanistic explanations. The central hypothesis evaluated here is that orthosteric sites are governed by steep energetic funnels that enforce unimodal convergence toward a unique native pose, whereas allosteric sites reside on flatter or degenerate landscapes that permit multiple geometrically distinct solutions with comparable interaction topologies. We argue that this energetic degeneracy drives systematic underperformance across diverse AI architectures and underlies the intrinsic difficulty of allosteric ligand-binding prediction. This approach addresses a central question raised by our benchmarking results: which features of allosteric sites render them resistant to accurate prediction, and, conversely, what features of orthosteric sites make them so reliably detectable? To answer this question, we employed local frustration analysis, an energy-landscape-theory framework that quantifies the energetic optimality of native interactions relative to large decoy ensembles.

This analysis provides complementary residue-level descriptors of (1) conformational frustration, which reports on local landscape topography relevant to pose placement, and (2) mutational frustration, which reflects evolutionary constraint encoded in sequence and thus informs pocket detectability. The frustration analysis was explicitly designed to mirror the dual prediction task faced by AI models. Our analysis separately interrogates (1) the intrinsic energetic organization of binding sites in the apo state, which governs detectability, and (2) the energetic reorganization induced by ligand binding, which governs geometric convergence. By comparing ligand-free (apo). and ligand-bound (holo) states across orthosteric and allosteric complexes, we identify which energetic features are inherent to the binding site itself—present in both the unbound and bound states—and which arise specifically upon ligand binding. This comparison is critical because it allows us to distinguish between two alternative explanations for the observed performance gap. If orthosteric sites are already enriched in minimal frustration in the apo state, their high detectability would reflect a pre-existing energetic feature. Conversely, if minimal frustration emerges only upon ligand binding, then the directional signal that guides AI models is induced rather than intrinsic. The same logic applies to allosteric sites: persistent neutral frustration in both apo and holo states would indicate that the lack of a directional signal is an inherent property of the site, not a consequence of ligand absence. This comparison thus provides a direct test of whether the energetic organization of binding sites is static or ligand dependent and whether the resulting frustration signatures can explain the differential performance of AI models.

**Figure 4.**
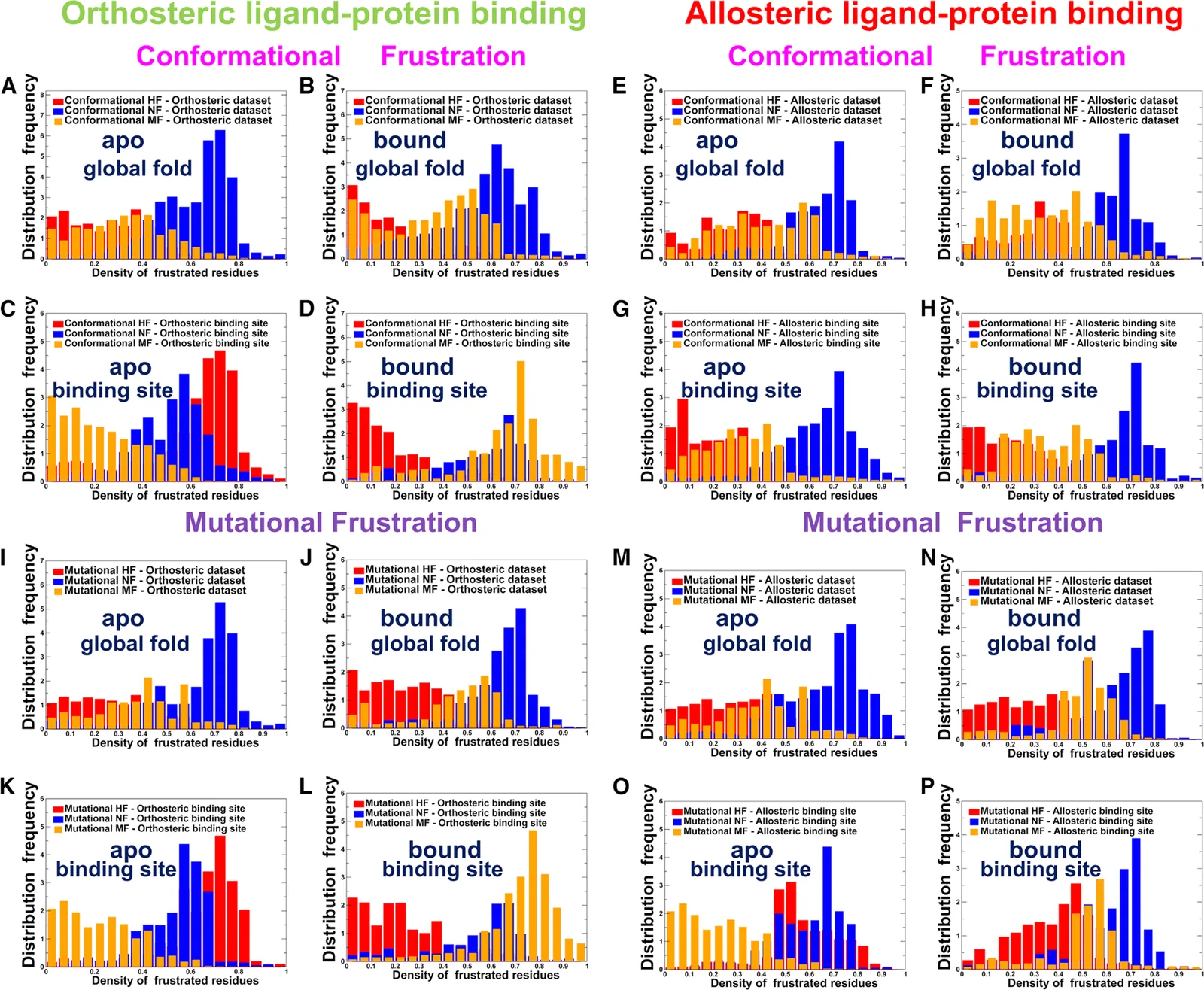
Frustration dichotomy encodes the energetic grammar of orthosteric versus allosteric binding sites (A–D) Conformational frustration profiles for orthosteric complexes. (A) Global fold, apo state. (B) Global fold, holo state. (C) Binding site, apo state. (D) Binding site, holo state. Orthosteric sites undergo pronounced frustration quenching upon ligand binding: highly frustrated contacts (red bars, *Z* < −1.0) decrease from 28% in the apo state to 8% in the holo state, while minimally frustrated contacts (orange bars, *Z* > 0.78) increase from 31% to 64%. Global fold frustration remains largely unchanged. (E–H) Conformational frustration profiles for allosteric complexes. (E) Global fold, apo state. (F) Global fold, holo state. (G) Binding site, apo state. (H) Binding site, holo state. In contrast to orthosteric sites, neutral frustration (blue, bars −1.0 ≤ *Z* ≤ 0.78) dominates both apo (71%) and holo (68%) allosteric sites, with no systematic shift upon ligand binding. (I–L) Mutational frustration profiles for orthosteric complexes. (I) Global fold, apo. (J) Global fold, holo. (K) Binding site, apo. (L) Binding site, holo. Orthosteric sites show strong enrichment of minimal mutational frustration (79%, orange) upon ligand binding. (M–P) Mutational frustration profiles for allosteric complexes. (M) Global fold, apo. (N) Global fold, holo. (O) Binding site, apo. (P) Binding site, holo. Allosteric sites remain dominated by neutral mutational frustration (58%, blue) in both states. Color coding: highly frustrated (HF) distributions are shown in red-filled bars. Neutrally frustrated (NF) distributions are in blue-filled bars. Minimally frustrated (MF) distributions are shown in orange-filled bars.

We first examined conformational frustration across entire protein folds to establish a global baseline. Orthosteric and allosteric complexes displayed nearly identical global frustration profiles in both apo and holo states (Figures 4A and 4B for orthosteric; Figures 4E and 4F for allosteric). In all cases, minimally frustrated residues dominated the hydrophobic core (~45%–55%), highly frustrated residues localized to functional loops and interfaces (~15%–20%), and neutral frustration characterized surface-exposed and flexible regions (~30%–40%). These distributions were invariant between apo and holo states and did not differ between orthosteric and allosteric complexes, demonstrating that the predictive dichotomy does not originate from global fold organization. Instead, it must arise from the energetic architecture of the binding sites themselves and from how those sites respond to ligand binding. In the apo state, orthosteric binding sites are enriched in highly frustrated contacts as ~28% of binding-site residues are highly frustrated compared to ~12% in the global fold (Figure 4C). The remaining binding-site residues are distributed between neutral (41%) and minimal (31%) frustration. Upon ligand binding, orthosteric sites undergo pronounced frustration quenching, generating the steep energetic funnels required for geometric convergence. In the holo state, the fraction of minimally frustrated residues increases from 31% to 64%, while highly frustrated contacts decrease from 28% to 8% (Figure 4D). Neutral frustration correspondingly decreases from 41% to 28%. Importantly, this reorganization is localized to the binding site, while global fold frustration remains essentially unchanged (Figures 4A and 4B).

Thus, ligand binding actively reshapes the local energy landscape rather than simply occupying a pre-existing cavity. More than one-quarter of binding-site residues transition from strained or ambiguous energetic states into optimally stabilized interactions. This frustration quenching generates a strong directional gradient in conformational space—a deep energetic funnel that guides sampling toward a unique native geometry. Critically, this directional signal is created by ligand binding itself and is absent in the apo structure. AI models trained predominantly on holo complexes and optimized to detect stabilization patterns are therefore ideally positioned to exploit these ligand-induced funnels for precise pose placement at orthosteric sites. Mutational frustration profiles reinforce the prevalence of neutral frustration for apo and holo forms of the global protein folds (Figures 4I and 4J) while revealing ligand-induced frustration quenching in orthosteric binding regions (Figures 4K and 4L). Orthosteric binding sites are strongly enriched in minimally frustrated residues, with 79% of binding-site positions classified as minimally frustrated in the holo state (Figures 4K and 4L).

To further validate that the frustration signatures observed in the original allosteric benchmark (Figure 4) generalize to independently curated allosteric datasets, we performed local frustration analysis on the ASD-derived test set and the non-redundant PLA set. The same methodology described in the methods section (conformational and mutational frustration, apo vs. holo states, global vs. binding-site regions) was applied. Tables S12–S15 mirror the quantitative data shown in Figure 4 for the original benchmark. The results showed that allosteric binding sites in both ASD and PLA datasets are dominated by neutral frustration (~65%–75%) in both apo and holo states, with no systematic frustration quenching upon ligand binding (Tables S14 and S15). This stands in stark contrast to orthosteric sites (Figure 4) where minimal frustration increases from ~30% to ~65% upon binding.

To move beyond qualitative correlation, we also performed several additional quantitative analyses. We computed, for each residue in each complex, the local frustration density (fraction of minimally frustrated contacts within 5 Å) and compared it to the model’s per-residue confidence score (predicted local distance difference test [pLDDT] for AF3/Chai-1; analogous metrics for other models). For orthosteric complexes, there is a moderate to strong positive correlation between local minimal frustration and model confidence (ρ ≈ 0.44–0.55). For allosteric complexes, the correlation is near zero (ρ ≈ 0.05–0.12) and not statistically significant for most models (Table S16). This quantitative dissociation supports the interpretation that models confidently assign high scores where the landscape is minimally frustrated for orthosteric ligand binding but cannot reliably gauge accuracy where the landscape is neutrally frustrated dominated in allosteric ligand binding.

To ensure that the frustration-success link is not driven by a single pocket or by pocket-level redundancy, we also conducted a leave-one-pocket-out analysis on the non-redundant PLA set (182 unique pockets, each represented by its highest-resolution structure). For each iteration, one pocket was held out, the frustration-based logistic regression model was retrained on the remaining 181 pockets, and success (RMSD <2 Å for AF3 oracle) was predicted for the held-out pocket. The procedure was repeated for all 182 pockets. The obtained leave-one-pocket-out accuracy of 82% (AUC = 0.84) indicated that the relationship between frustration features and prediction success is robust and not dependent on any individual pocket. These analyses provide strong quantitative evidence that the frustration signature is a stable, pocket-intrinsic property that generalizes across ligand instances and across pockets.

It is important to note, however, that a limitation of the frustration analysis is its correlative nature. While the quantitative links we have established using per-residue confidence correlation and leave-one-pocket-out validation) strengthen the case for a mechanistic role of the energy landscape, they do not prove causality. To test causality more directly, future work should (1) introduce point mutations that convert a minimally frustrated orthosteric pocket into a neutrally frustrated one and test whether prediction accuracy drops; (2) conversely, engineer minimal frustration into an allosteric pocket (e.g., by stabilizing key contacts) and test whether prediction accuracy improves; (3) incorporate MD simulations to directly compute the free energy landscape of binding and compare funnel depth and barrier heights to frustration metrics and prediction outcomes. Such experiments would move beyond correlation toward causal validation of the energy-landscape hypothesis.

### Persistent neutrality and evolutionary constraints prevent energetic convergence at allosteric sites and reinforce prediction dichotomy

Allosteric binding sites in the apo state are dominated by neutral frustration, with 71% of residues classified as neutrally frustrated (Figure 4G). Highly frustrated contacts are rare, and minimally frustrated residues are sparse. This persistent neutrality means that allosteric binding regions lack distinctive energetic landmarks in the frustration landscape. In stark contrast to orthosteric complexes, allosteric binding sites exhibit no analogous frustration quenching upon ligand binding (Figure 4H). In both apo and holo states, neutral frustration dominates the binding region (71% and 68%, respectively), with minimally frustrated residues remaining modest (21% in apo, 24% in holo) and highly frustrated contacts rare (Figures 4G and 4H). The energetic frustration distribution of allosteric binding sites may be partially redistributed but displays persistent neutrality patterns upon ligand binding. As a result, multiple putative allosteric pockets can satisfy similar interaction topologies producing energetic degeneracy and preventing reproducible pocket detection and ligand-pose convergence. Mutational frustration reveals a parallel divergence in evolutionary organization that compounds the prediction challenge of allosteric binding. Neutral mutational frustration not only is persistent for the global protein folds of allosteric complexes (Figures 4M and 4N) but also dominates the frustration patterns of allosteric binding regions in both apo and holo states (Figures 4O and 4P) with only 34% of residues being minimally frustrated. This evolutionary permissiveness reflects the functional logic of allostery, which requires conformational and evolutionary plasticity to mediate large structural rearrangements through moderate energetic barriers. Multiple geometrically distinct poses can satisfy similar contact topologies, and our findings suggest that coevolutionary signals alone could not provide the directional gradient needed to repeatedly converge to a single and accurate binding solution. Indeed, a recent pioneering study showed that structural pattern learning may have significantly greater effect on protein fold predictions than information inferred from coevolutionary couplings.^87^

Coevolutionary coupling has been shown to identify functional sectors that are essential for both protein stability and binding.^88–90^ In orthosteric pockets, these coevolutionary signals are especially strong and consistent, providing reliable statistical patterns that AI architectures are specifically trained to detect, which can explain why models consistently achieve high accuracy for orthosteric pose prediction. Importantly, allosteric sites are not necessarily devoid of coevolutionary coupling. Allosteric proteins contain sparse, physically contiguous sectors of coevolving residues that mediate long-range communication and regulation.^88–90^ However, these coevolutionary signals serve a different function as they encode allosteric communication pathways rather than a unique allosteric ligand-binding geometry. Thus, allosteric ligand binding lacks not any coevolutionary signal but, rather, the tight, geometrically constrained coupling that would enforce a unique pose—a limitation that is consistent with both the frustration framework and structural memorization effects.

To summarize, we found that orthosteric binding sites undergo frustration quenching upon ligand binding, transitioning from elevated apo-state frustration to minimal frustration upon ligand binding and creating steep landscape funnels serving as unambiguous signals for AI convergence. In contrast, allosteric binding can preserve neutral frustration in both apo and holo states that obscures structural and evolutionary patterns that AI models are trained to detect, thus underlying the biophysical logic behind model-independent performance gap.

### Structural organization of frustration landscapes encodes binding-site predictability

**Figure 5.**
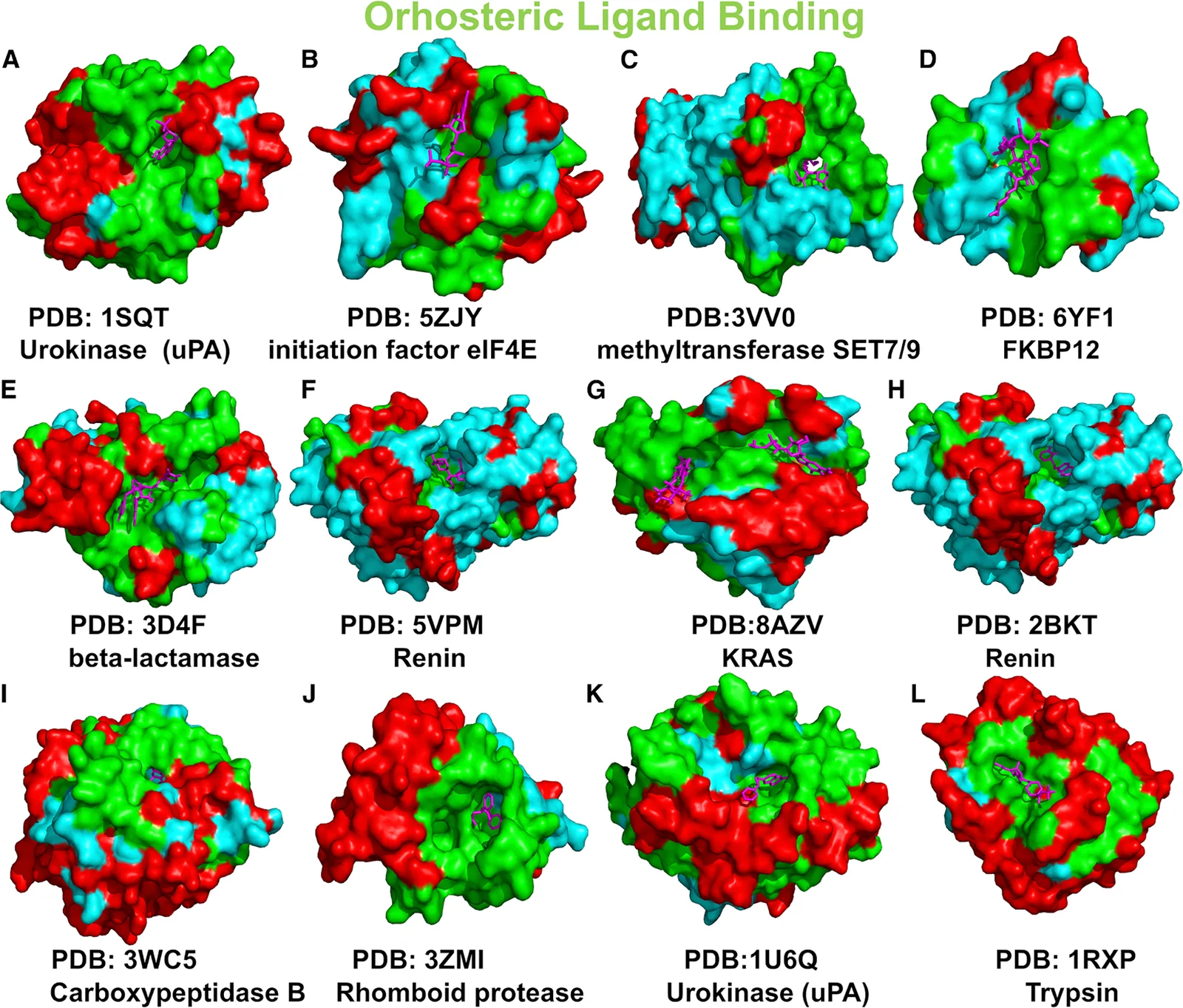
Structural maps of orthosteric complexes reveal extensive minimally frustrated networks across diverse protein families (A–L) For a representative panel of orthosteric ligand-protein complexes, MF regions are shown as green semi-transparent surfaces; NF regions are shown as red surfaces. The bound ligands are shown as magenta-colored solid sticks. Orthosteric pockets are uniformly small, deeply buried clefts. MF regions extend well beyond the immediate binding pocket into neighboring structural elements.

The quantitative frustration patterns establish a clear energetic dichotomy between orthosteric and allosteric binding sites, but aggregate distributions cannot reveal how these differences manifest in three dimensions. Mapping frustration states onto representative protein structures provides this spatial context, revealing consistent architectural principles that govern pocket architecture and directly inform the dual prediction challenge of pocket identification and pose placement. Orthosteric complexes exhibit a conserved spatial architecture that transcends individual protein families (Figure 5). Across the representative panel, binding pockets are uniformly compact and deeply embedded within the protein core, typically at domain interfaces or within conserved catalytic clefts. Critically, minimally frustrated residues form extensive, spatially contiguous networks. In each complex, the minimally frustrated region envelops the binding pocket in a continuous shell that extends 8–12 Å into the surrounding structure, creating broad, cooperatively stabilized zones. This spatial continuity is the structural manifestation of the ligand-induced frustration quenching where 64% of binding-site residues transition to minimal frustration upon ligand engagement. The resulting landscape provides a distinctive energetic signal to AI models for unambiguous pocket identification and an extended directional funnel that guides conformational sampling toward a unique native geometry.

Residue-level distribution profiles of conformational frustration (Figure S7) and mutational frustration (Figure S8) for a representative panel of orthosteric complexes corroborate this organization, revealing dense clusters of minimally frustrated residues at positions surrounding the binding pocket, which is the recurrent biophysical signature that pattern-recognition architectures are optimized to detect. Notably, the spatial extent of these networks correlates with prediction confidence: systems with the most extensive minimal frustration shells correspond to those where AF3 and Chai-1 achieve sub-angstrom accuracy.

**Figure 6.**
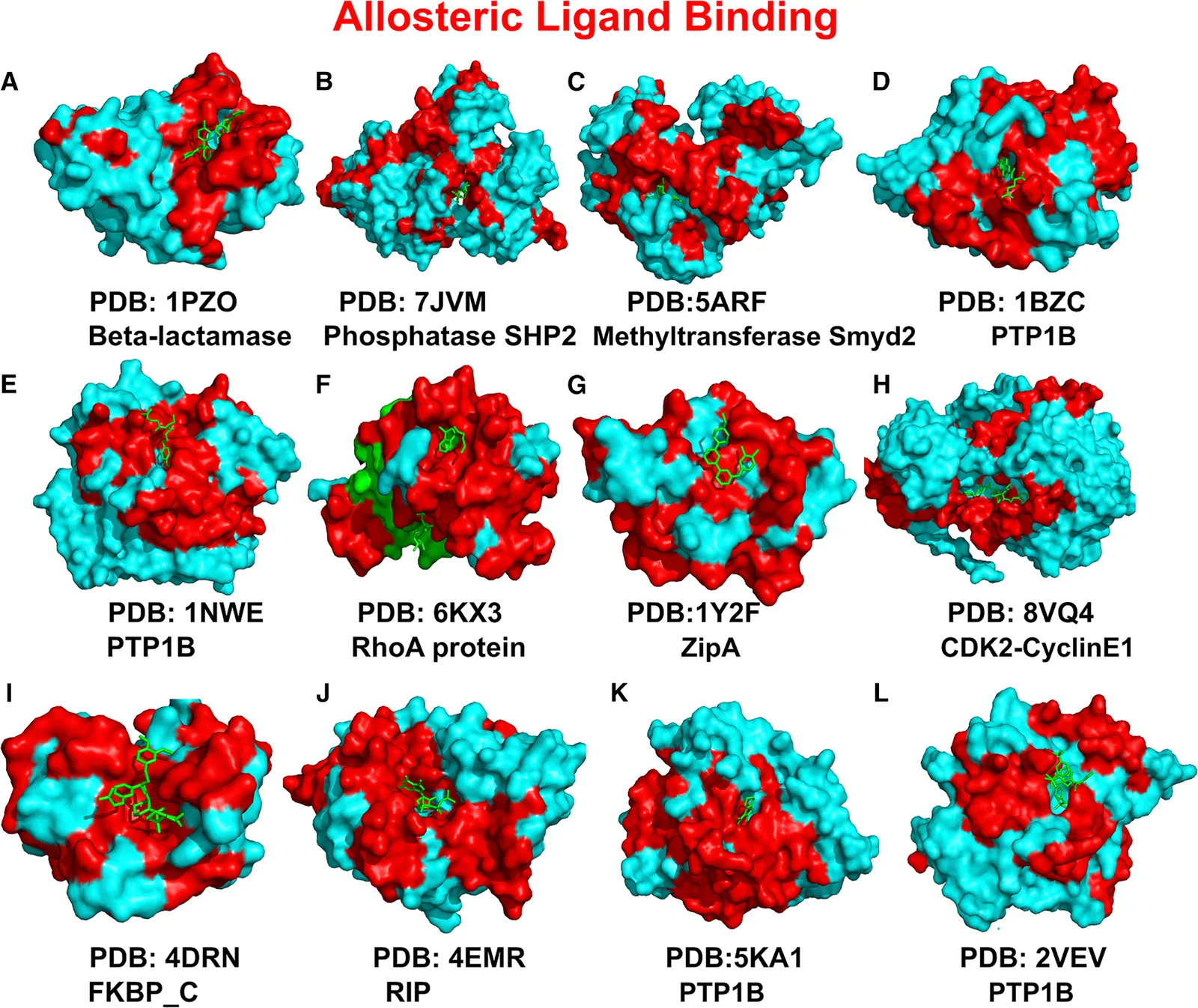
Structural maps of allosteric complexes show neutral frustration dominating binding-site environments across diverse regulatory architectures For a representative panel of allosteric complexes, NF regions (shown in red surfaces) dominate both global folds and binding-site regions. Allosteric pockets are embedded within extensive NF zones, with only isolated, disconnected patches of MF regions (shown in green surfaces). The bound allosteric ligands are shown in green-colored sticks, and these ligands occupy predominantly NF regions.

Allosteric complexes display a distinct spatial grammar that holds across diverse regulatory architectures (Figure 6). The representative panel includes buried allosteric pockets, surface-exposed allosteric grooves, and cryptic sites that form upon ligand binding. Despite this architectural diversity, the frustration signature is remarkably consistent. Neutral frustration dominates throughout the protein fold, including directly within binding regions. Minimal frustration appears only as isolated, spatially disconnected patches that are scattered within expansive neutrally frustrated zones. In deeply buried allosteric pockets that superficially resemble orthosteric sites, the frustration maps reveal the critical distinction: these pockets lack the extended minimal frustration networks characteristic of their orthosteric counterparts. The surrounding region remains predominantly neutral, with only sporadic minimal frustration at key anchor points. For surface-exposed sites, neutral frustration pervades the entire binding interface, with minimal frustration relegated to distal structural elements uninvolved in ligand recognition. Residue-level distribution profiles of conformational (Figure S9) and mutational frustration (Figure S10) for a representative panel of allosteric complexes illustrate diversity of frustration organization, showing sparse, non-contiguous minimal frustration clusters and pervasive neutral mutational frustration. The central conclusions of this study are that orthosteric binding sites are enriched in minimally frustrated residues and allosteric binding sites are dominated by neutral frustration, and these findings are based on aggregate statistics over thousands of complexes (Figure 4). At the same time, the residue-based frustration patterns across the entire protein sequence are shown for a set of representative complexes to highlight the intrinsic diversity and variability of frustration patterns on a per-residue basis. In particular, the presence of minimal frustration peaks in allosteric profiles often reflects residues outside the allosteric pocket (e.g., orthosteric sites or stable cores) and does not contradict the pocket-focused statistical analysis. The binding pocket constitutes only a small fraction of the residues displayed, but, nonetheless, the residue-based frustration profiles highlighted absence of spatially coherent minimal frustration networks for allosteric complexes, which may have consequences for prediction performance. Even when allosteric pockets are approximately localized, the absence of extended minimally frustrated residue networks surrounding the binding site can impede formation of broad energetic funnels and robust geometric convergence to a unique ligand-binding pose. The isolated minimal frustration patches may provide weak local constraints, anchoring specific interactions without specifying global orientation, but they are insufficient to resolve the full three-dimensional arrangement.

Together, these spatial features encode the dual challenge of allosteric prediction. Orthosteric landscapes broadcast a spatially coherent, evolutionarily reinforced signal through connected minimal frustration networks—a signature that specifies a unique solution by providing both location and orientation. Allosteric landscapes, by contrast, remain neutrally frustrated, energetically degenerate, and evolutionarily permissive in both apo and holo states, withholding the directional signals AI architectures require. Prediction accuracy thus becomes a quantitative reporter of energy-landscape architecture, transforming model performance from a descriptive statistic into mechanistic insight into the physical grammar of allosteric recognition.

## DISCUSSION

The integration of large-scale AI benchmarking with energy-landscape analysis reveals a strong association between binding-site predictability and biophysical organization. Across five state-of-the-art co-folding architectures—each embodying distinct design principles—the pattern remains consistent. Orthosteric predictions achieve near-experimental accuracy with unimodal convergence, while allosteric predictions collapse systematically into broad, flat distributions. The marked degradation across models instead points to a common underlying physical property of allosteric sites that current architectures cannot overcome and suggests an additional rationale to explanations rooted solely in limitations of training data or architectural idiosyncrasies.

Although it is tempting to interpret the observed dichotomy and our findings as primarily a signature of biophysical allosteric grammar underpinned by energy-landscape differences, we must consider important complementary explanations raised by recent studies^86,87^ that provided compelling evidence of structural memorization and revealed that predictions of AI models can be largely determined by memorization of ligand binding from training data. It is also plausible that the observed underperformance on allosteric complexes is driven, at least in part, by dataset distribution bias: the vast majority of holo training structures correspond to orthosteric binding, whereas allosteric complexes are comparatively rare and heterogeneous. Our data confirmed that structural memorization contributes to ligand-binding prediction for allosteric complexes with statistically significant structural coverage.

Rather than treating memorization and energy-landscape reasoning as mutually exclusive, we propose that these factors may constitute major complementary drivers of prediction performances by current AI co-folding models. In this synergistic interpretation, AI models can successfully learn the low-frustration, deeply funneled orthosteric landscapes because these are both evolutionarily conserved and densely sampled in training. For allosteric sites, which lie in neutrally frustrated regions of the energy landscape that are evolutionarily plastic and sparsely represented in training, models default to pattern matching based on memorization of training-set examples and fail to learn a robust, generalizable energy function that discriminates accurate from inaccurate poses. This synthesis is supported by several lines of evidence. First, independent energy-landscape analyses have shown that orthosteric binding sites exhibit minimal local frustration that creates a dominant energetic funnel, whereas allosteric pockets are predominantly neutrally frustrated and associated with conformational plasticity.^25,91^ Second, our dual-framework interpretability pipeline directly links prediction outcomes to frustration landscapes, showing that orthosteric binding quenches frustration while allosteric sites retain neutral frustration in both apo and holo states. Even if an allosteric site were abundantly represented in training, the flat frustration landscape would still permit multiple geometrically distinct solutions with comparable interaction topologies, preventing unique convergence—a prediction supported by our coverage-stratified analysis. Conversely, even if a site were rarely seen in training, a steep energetic funnel (as in orthosteric sites) could still guide the model toward the correct geometry through physical reasoning. Thus, memorization alone cannot rescue allosteric prediction, requiring the underlying energy landscape to provide a directional signal. Our frustration analysis provides a biophysically grounded explanation for why such a signal is absent in allosteric sites and present in orthosteric sites. In this light, AI prediction accuracy may also serve as a quantitative reporter of underlying biophysical organization. Orthosteric sites broadcast strong signals through ligand-induced funnels, evolutionary constraints, and spatially coherent minimal frustration networks. Allosteric ligand binding is characterized by persistent neutral frustration, mutational permissiveness, and conformational heterogeneity that collectively can conceal a unique solution and challenge the memorization patterns that AI models are trained to decipher.

A critical distinction must be drawn between predicting the binding mode of a ligand at a defined allosteric site—the task we have systematically benchmarked—and predicting allosteric regulation more broadly, which encompasses ligand-induced topological rearrangements (e.g., domain reorientation, fold switching, quaternary structure changes), long-range signal propagation, and shifts in population ensembles. Our study is deliberately focused on the former, narrower problem. Importantly, the central claim of our paper that current AI co-folding models produce geometrically inaccurate poses for allosteric ligands while preserving native contact topology (high QS score) does not depend on whether the protein undergoes a large or small conformational change. The input to each model is the apo protein sequence (or structure, depending on the model) and the ligand. The model must place the ligand in the correct holo pocket. Even when the holo pocket is formed by a substantial backbone movement, the prediction task remains well defined. Nevertheless, for allosteric systems that involve extreme topological changes (e.g., fold switching, large domain movements >8–10 Å, or order-disorder transitions), the prediction challenge is compounded by the need to model alternative protein conformations—a task for which current co-folding models are known to perform poorly even in orthosteric cases. The frustration analysis, extended beyond the local binding pockets and involving dynamic ensembles, might inform the study of allosteric communication. Future research should integrate these global frustration metrics with AI prediction outcomes to ask whether models also struggle with allosteric systems that require backbone rearrangements and whether the energetic signatures of those rearrangements can be anticipated from sequence or structure alone.

The systematic limitations of AI co-folding models on allosteric complexes, coupled with the energetic concealment revealed by frustration analysis, resonate with emerging recent work interrogating the limits of AI for protein-ligand prediction. Three complementary studies released very recently, each addressing distinct aspects of co-folding performance, provide striking conceptual alignment with our findings and provide compelling external validation for the biophysical framework we have established.^92–94^ In a prospective evaluation of 557 SARS-CoV-2 Mac1-ligand complexes, Kim et al. demonstrated that, while AF3, Boltz-2, and Chai-1 each reproduced >50% of poses within 2 Å RMSD, all models failed to recapitulate essential protein conformational changes required for ligand binding.^92^ This inability to model conformational adaptation directly parallels the systematic failure we observe on allosteric complexes, where structural plasticity is not incidental but definitional. The PLINDER resource introduced by Durairaj et al. addresses the foundational challenge of rigorous, leakage-minimized benchmarking, providing over 449,000 protein-ligand systems with multi-level similarity metrics that enable realistic assessment of model generalization.^93^ Their recognition that diverse inference scenarios, including novel pockets and protein-ligand combinations, require specialized stratified test sets directly supports our demonstration that allosteric sites represent a fundamental generalization challenge rather than a simple performance decrement. The diversity of allosteric site architectures suggests that different allosteric mechanisms may present different prediction challenges; larger datasets with finer mechanistic annotation could enable stratification of allosteric sites by their frustration signatures and corresponding prediction outcomes. Most directly, Zhang et al. benchmarked co-folding methods on 218 covalent complexes and found that, while models achieved superior accuracy on familiar systems, performance declined markedly for novel pocket-ligand pairs,^94^ which is a pattern mirroring the allosteric performance gap we report in this study.

Collectively, these independent observations reinforce our central thesis: the allosteric blind spot is not a mere memorization failure but a diagnostic of a biophysical grammar where neutral frustration conceals unique solutions. While our controls explored underlying reasons behind memorization and training bias, we acknowledge that definitive causation cannot be established from correlational analyses alone. The memorization perspective remains a valid complementary explanation. To rigorously distinguish memorization from learned energetics, future work will require (1) time-split allosteric benchmarks, (2) mutagenesis-driven prediction tests that systematically alter allosteric pocket residues and evaluate whether models can predict the resulting changes in binding mode—a powerful approach to probe memorization versus generalization, and (3) fine-tuning or retraining of co-folding models on balanced orthosteric-allosteric datasets to directly assess whether denser sampling of allosteric complexes improves geometric accuracy. Another direct test of the memorization versus biophysical constraint hypothesis would be to occlude the orthosteric site during prediction—for example, by masking its residues or by providing the model with an apo structure where the orthosteric pocket is already occupied by a dummy ligand—and then re-evaluating allosteric prediction. Under a pure memorization model, occluding the orthosteric site might force the model to rely on weaker allosteric training signals and potentially improve allosteric pose accuracy. Under our frustration-based framework, however, we predict that occluding the orthosteric site would have less significant effect, because the fundamental limitation is the absence of a unique energetic funnel at the allosteric site itself, not competition with a memorized orthosteric solution.

Our study establishes a principled, biophysics-informed explainable-AI framework that transforms the allosteric blind spot from a limitation into an insight. By synthesizing memorization and energy-landscape grammar as complementary pillars, we provide both a diagnostic tool for evaluating next-generation models and a potential roadmap for developing energy-aware architectures capable of navigating allosteric regulatory landscapes.

## METHODS

### Orthosteric datasets

The MDT test set served as the primary orthosteric reference, comprising 412 complexes.^39^ Structural challenge was enforced by requiring AlphaFold-predicted apo-pocket deviation from experimental holo-conformation >2.0-Å RMSD or pocket LDDT <0.80. Ligands were restricted to drug-like molecules (200–650 Da), excluding fragments and macrocycles,^39^ with no more than 10 complexes from any single study. The datasets were filtered to include only complexes deposited in the Protein DataBank after 2020, ensuring they post-date model training sets. Complexes with >30% sequence identity to training data were excluded (verified via PDB70 and MMseqs2 clustering). To expand statistical power, we incorporated the PLOC subset from a comprehensive structural dataset^85^ containing 1,863 structures with 2,325 binding pockets and 1,647 unique ligands binding to orthosteric sites across 76 Pfam families. Combined with MDT, the comprehensive orthosteric reference totaled 2,275 complexes.

### Allosteric datasets

Kinase-specific allosteric complexes were derived from the Kinase Conformation Resource (KinCoRe)^81,82^ a geometry-driven classification system using DFG motif and αC-helix conformation. Type III inhibitors bind adjacent to the ATP site (distance >6.0 Å from hinge region, minimum three contacts with back-pocket residues). Type IV allosteric inhibitors are distal allosteric inhibitors that bind to truly distal sites, far from the catalytic machinery. Type IV allosteric modulators can bind to any kinase state, as they do not interact with the active site motifs directly. According to KinCoRe criterion, type IV allosteric ligand must be >6.5 Å away from both the hinge region and the C-helix-Glu(+4) residue. From this resource, we extracted 217 type III/IV complexes, augmented with a curated X-ray crystallography repository^83^ containing 136 allosteric kinase inhibitors (resolution ≤ 3.5 Å, MW ≥ 250 Da, no primary site occupancy). Allosteric kinase modulators target a wide range of locations, including the well-characterized myristoyl pocket in the C-lobe (type IV), the back pocket adjacent to the ATP site (type III), and several less-conserved surface grooves The kinase allosteric dataset totals 353 complexes. To ensure comparability with the orthosteric benchmark, we have additionally applied identical temporal and sequence-similarity filters: only structures deposited after 2020 were retained, and any complex with ≥30% sequence identity to any structure in the training sets (as determined by MMseqs2 clustering against PDB70) was removed. Additionally, we applied a pocket-level similarity filter: allosteric pockets with ≥40% residue identity to a pocket in the training set were excluded. After these filters, the kinase allosteric dataset comprised 321 high-confidence complexes.

To ensure biologically relevant chain and ligand selection, we relied on the pre-curated annotations provided by KinCoRe. For each PDB entry, KinCoRe defines the biological assembly (not the asymmetric unit), which contains only the functionally relevant copies of each polypeptide chain. The ligand set is derived from the biologically relevant small molecules listed in KinCoRe; only ligands satisfying the KinCoRe classification criteria and directly bound to the protein are retained. The following quality-control checks are included: (1) only structures with resolution ≤ 3.0 Å, (2) exclusion of ligand atoms with occupancy < 0.5, (3) use of only the highest-occupancy conformer for alternate conformations, and (4) manual inspection of a random subset of 50 complexes to confirm chain composition matches the biological assembly.

To capture allosteric diversity, we also incorporated the PLA subset,^66^ containing a total 1,613 structures with 1,830 binding pockets and 1,277 unique ligands spanning 83 Pfam families across >500 organisms. All complexes were experimentally validated as authentic allosteric binders with sites spatially distinct from orthosteric pockets. A total of 1,830 binding pockets reported in the PLA subset represent the total number of complexes but not distinct allosteric sites. To address this. we further processed the PLA subset to distinguish total complexes from genuinely distinct allosteric pockets. Using UniProt IDs, pocket residue composition, and geometric clustering (Cα RMSD ≤2.0 Å), we identified 182 unique allosteric pockets spanning 115 distinct UniProt entries. For each unique pocket, we selected the highest-resolution representative structure for primary benchmarking. This yields a non-redundant allosteric benchmark that eliminates multiple counting of the same pocket bound to different ligands. We further implemented a pocket-level similarity filter for allosteric complexes: any allosteric pocket with ≥40% residue identity to a pocket in the ASD training set was excluded. This ensures that the performance gap we report cannot be attributed to uneven data-leakage protection. After this additional filtering, the allosteric dataset comprises 2,184 high-confidence orthosteric complexes and 503 filtered allosteric complexes.

In addition, to further validate our findings on an independent, community-standard resource, we curated a high-confidence allosteric test set from ASD dataset^66^ containing PDB codes, UniProt IDs, allosteric residue names, and chain IDs (ASD_Release_202309_AS.tar.gz, release v.5.1). We followed a recently validated selection protocol^84^ in which, of the list of 3,102 ASD protein entries, we selected human proteins with modulator_class = “Lig” (small-molecule sites, 206 proteins). We further curated the ASD subset to allosteric complexes with valid allosteric ligand cavity annotations and complete site mapping. This quality-control and site-deduplication step reduced the set to 141 proteins spanning 182 unique sites. This approach ensures that the ASD subset is (1) high confidence with experimentally validated allosteric ligands and well-defined binding residues, (2) non-redundant (single chain, length filtered), and (3) comparable to the AF2BIND benchmark^84^ allowing direct cross-validation.

### AI models

Five state-of-the-art architectures were benchmarked following protocols adapted from PoseBusters.^31^ All models received identical inputs: protein sequences from PDB structures, ligands in SMILES format. For each complex, 25 independent predictions were generated (10 recycling steps, 200 diffusion steps, five seeds, five diffusion samples per seed) on NVIDIA A100 40GB GPUs. The ligand-protein chain-pair iPTM score was used for ranking. Scripts are available in the accompanying code repository. AF3 employs a diffusion-based architecture operating on a unified “cloud of atoms,” simultaneously diffusing protein and ligand coordinates.^30^ Predictions were generated with templates enabled (default) and without templates (AF3-NT). Confidence metrics include pTM, ipTM, per-atom pLDDT, and predicted aligned error (PAE). Protenix is an open-source implementation of AF3 using unified tokenization of polymers and small molecules while maintaining architectural fidelity.^34^ Boltz-2 extends diffusion-based co-folding with affinity and binder-classification heads trained on experimental and MD-derived data.^35^ It incorporates prediction heads for affinity estimation and binder classification, using confidence-guided sampling to prioritize predictions with high internal consistency. Chai-1 uses evolutionary scale modeling (ESM)-2 PLM embeddings with paired attention mechanisms, incorporating evolutionary information directly into structure prediction.^38^ Predictions were made with the ESM embedding option enabled. DynamicBind employs an SE(3)-equivariant diffusion graph network that iteratively adjusts receptor structure to accommodate the ligand.^39^ Training involves progressively distorting holo structures with noise and learning the reverse diffusion process that recovers native conformations. During inference, DynamicBind generates 20 candidate trajectories per target, scored using a composite metric combining LDDT scores and predicted binding affinities.

### Prediction selection and confidence scoring

For each complex, we generated 25 independent predictions (5 seeds × 5 diffusion samples) per model, except for DynamicBind (20 trajectories). For the oracle analysis (upper bound), we selected the single prediction with the lowest ligand pose RMSD relative to the experimental structure. We evaluated two additional strategies: (1) model-selected top-ranked—the single prediction with the highest native confidence score (ipTM for AF3, Protenix, Boltz-2, and Chai-1; composite LDDT-affinity score for DynamicBind); and (2) average (mean-of-N)—the mean RMSD over all 25 predictions, which provides a conservative lower bound. We performed this analysis on the original benchmark as well as on the ASD and non-redundant PLA datasets.

### Evaluation metrics and alignment pipeline

All predicted protein structures were superimposed onto experimental references using TM-align^95^ for sequence-independent structural alignment. TM score quantifies global structural similarity on a scale of 0–1, where values > 0.5 indicate the same fold and values > 0.9 indicate near-identical backbone conformation. TM-scores were computed using TM-align after optimal superposition of predicted and experimental structures. Several ligand RMSD tools such as LigRMSD,^96^ DockRMSD,^97^ and spyRMSD,^98^ which are commonly applied in docking evaluations, were initially employed but large-scale pipeline execution of diverse AI models require clean ligand definitions, consistent atom ordering, and stable residue naming conventions, which appear to be highly heterogeneous and inconsistent across different AI models outputs. OpenStructure framework was adopted in the final protocol, which allows direct control over structure alignment, ligand extraction, and RMSD calculation within a single framework.^99^ We calculated the ligand-pose RMSD with the OpenStructure toolkit (OST) using the Symmetry-Corrected RMSD Scorer (SCRMSDScorer), which enumerates symmetry-equivalent atom assignments and returns the lowest RMSD.^99^ The binding pocket was defined as all protein residues with at least one atom within 8 Å of any reference ligand atom. After defining the pocket, RMSD was calculated over the backbone atoms of these residues following the same global structural alignment used for ligand RMSD. QS score quantified recovery of native contact topology independent of pose geometry, measuring the fraction of native ligand-protein contacts (4.5 Å cutoff) correctly recovered. Local distance difference test for ligand-protein interfaces (LDDT-LP) evaluated preservation of local atomic distances within the binding pocket, with sensitivity to side-chain interactions. Ligand-centric confidence metrics included L-pLDDT (mean pLDDT over ligand atoms) and L-PAE (average PAE involving ligand atoms) for AF3/Chai-1, and ipTM for Boltz-2. Confidence-error correlation was assessed by mapping confidence scores against actual RMSD.

### Local frustration analysis

We explored local frustration analysis, which is a framework rooted in energy-landscape theory that quantifies local energetic conflicts arising from competing interactions within a folded protein.^71–76^ Crucially, the frustration analysis was performed as an independent, hypothesis-driven test. The frustration signatures were computed solely from the protein structures using established energy-landscape theory without any knowledge of the prediction outcomes. Two complementary frustration indices were computed for each residue-residue contact in the protein structure. Configurational (conformational) frustration assesses the sensitivity of a native contact to local structural perturbations. It is calculated by randomizing both residue identities and interatomic distances within the native contact geometry, generating an ensemble of 1,000 decoy contacts. The energy of the native contact ($E_{\mathrm{native}}$) is compared to the mean energy of the decoys ($\langle E_{\mathrm{decoys}}\rangle$) and its standard deviation ($\sigma \left(E_{\mathrm{decoys}}\right)$) to compute a Z score.

Mutational frustration evaluates the energetic optimality of a given residue-residue pair by comparing its interaction energy to that of all possible amino acid substitutions at the same positions, while the backbone conformation remains fixed. The same Z score formula is used, with decoys generated by mutating the residue pair to all 20 × 20 amino acid combinations (excluding the native pair). Both frustration indices were expressed as Z scores, defined as

(Equation 1) $$Z=\frac{E_{native}\;-\;\langle E_{decoys}\rangle }{\sigma \left(E_{decoys}\right)}.$$

Following well-established thresholds validated across protein systems, contacts are classified into three regimes: minimally frustrated (*Z* > 0.78), where the native interaction is significantly more favorable than decoy alternatives, indicating strong energetic optimization and evolutionary constraint; neutrally frustrated (−1.0 ≤ *Z* ≤ 0.78), where the native interaction is neither strongly favored nor disfavored, consistent with conformational plasticity and mutational tolerance; and highly frustrated (*Z* < −1.0), where the native interaction is significantly less favorable than decoy alternatives, reflecting local strain or conflict. To obtain residue-level frustration profiles, we compute for each residue the local density of frustrated contacts within a 5 Å radius. Specifically, for a given residue *i*, we identify all residues *j* such that the Cα-Cα distance between them is ≤5 Å.

Among these contacts, we count how many are minimally, neutrally, or highly frustrated. The relative frustration density for a class is the number of contacts in that class divided by the total number of contacts within the 5-Å neighborhood. A residue is assigned a dominant frustration category if more than 50% of its interacting contacts within the most populated conformational ensemble belong to the same frustration class. To summarize, the workflow of the frustration analysis includes the following steps: (1) for each protein structure in the KinCoRe dataset, we extract all residue-residue contacts with Cα-Cα distance ≤10 Å (the default cutoff for frustration calculation); (2) for each contact, configurational and mutational *Z* scores are computed using the frustratometeR R package (version 2.0, https://github.com/proteinphysiologylab/frustratometeR); (3) each contact is then classified into one of the three frustration regimes using the *Z* score thresholds above; (4) contact-level classifications are converted to residue-level density by counting contacts within a 5 Å radius; and (5) we assign a dominant frustration category to each residue using the 50% majority rule. All calculations were performed on a high-performance computing cluster using the supercomputer implementation of frustratometeR.

### Quantification and statistical analysis

For each model, the best prediction per complex (lowest ligand-pose RMSD) was selected from generated models. Average RMSD values represent the mean of best-run RMSDs across all systems in each dataset. Statistical significance of differences between orthosteric and allosteric predictions was assessed using paired two-tailed *t* tests (*p* < 0.05) with Benjamini–Hochberg false discovery rate (FDR) correction.^100^ Residue-level frustration densities were compared using Mann–Whitney U test, with effect sizes quantified by Cliff’s delta.^101^

### Software implementation

All computations were performed in Python 3.10 using SciPy (v1.10) for hypothesis testing, stats models (v0.14) for multiple comparison correction, and custom scripts for effect size and correlation analysis. Visualizations were generated with Matplotlib (v3.7) and Seaborn (v0.12). The full evaluation pipeline, including all scripts for input preparation, model execution, output postprocessing, and statistical analysis, is available in our open-source repository with detailed documentation for community adoption and extension.

## RESOURCE AVAILABILITY

### Lead contact

Further information and requests for resources and materials should be directed to and will be fulfilled by the lead contact, Professor Gennady M. Verkhivker, verkhivk@chapman.edu.

### Materials availability

No materials were synthesized in this study.

### Data and code availability

- All unprocessed data and original code that the paper reports are freely available in an online repository that meets the criteria for digital longevity, implementation of FAIR? standards, and community support.
- All data have been deposited at Zenodo and are available at records (https://zenodo.org/records/18424406) and https://zenodo.org/records/20422508. The data are publicly available as of the date of publication at https://doi.org/10.5281/zenodo.18424405.
- All original code has been deposited at (https://github.com/Verkhivker-Research-Group/AI-CoFolding-Allostery-Benchmark) and is publicly available at https://doi.org/10.5281/zenodo.18424405 as of the date of publication. The new data and updated code and results are also available at https://github.com/Verkhivker-Research-Group/AI-CoFolding-Allostery-Benchmark-Revision/
- Any additional information required to reanalyze the data reported in this paper is available from the lead contact upon request.
- Data for this article, including description of data types, all data, software, and scripts, are freely available at the GitHub repository https://github.com/Verkhivker-Research-Group/AI-CoFolding-Allostery-Benchmark/tree/main, https://github.com/Verkhivker-Research-Group/AI-CoFolding-Allostery-Benchmark-Revision/and Zenodo archive https://zenodo.org/records/18424406.
- GitHub repository https://github.com/Verkhivker-Research-Group/AI-CoFolding-Allostery-Benchmark/tree/main and https://github.com/Verkhivker-Research-Group/AI-CoFolding-Allostery-Benchmark-Revision/contain information about orthosteric dataset and allosteric dataset. All scripts required to reproduce the predictions reported here are publicly available in the GitHub repository. The GitHub repository contains processed prediction outputs, evaluation results, figures, and scripts used in the study. The Zenodo archive https://zenodo.org/records/18424406 provides the datasets, processed prediction outputs, evaluation results, and scripts used in the study. Protein and ligand structures are not included in this archive. All structures are publicly available from the RCSB Protein DataBank (http://www.rcsb.org) and can be automatically downloaded using the provided scripts. The rendering of protein structures was done with UCSF ChimeraX package (https://www.rbvi.ucsf.edu/chimerax/) and Pymol (https://pymol.org/2/). All structures are publicly available from the RCSB Protein DataBank and can be automatically downloaded using the provided scripts.

## Supplementary Material

1

SUPPLEMENTAL INFORMATION

Supplemental information can be found online at https://doi.org/10.1016/j.xcrp.2026.103448.

## Highlights

- AI models reveal universal limitations in predicting allosteric ligand binding
- Physics-based explainable AI resolves the paradox of allosteric blind spots
- Neutral frustration in energy landscapes conceals allosteric patterns from AI models
- Explainable-AI framework unmasks biophysical grammar of allosteric drug discovery
