# Supplementary material for: Explainable AI reveals the allosteric blind spot in protein-ligand binding predictions
Source: Cell Rep Phys Sci. Author manuscript; Available in PMC 2026 Sep 11. (PMC13560746; doi:10.1016/j.xcrp.2026.103448)
Supplement: Supplementary file 1 [file NIHMS2205547-supplement-1.pdf]

Cell Reports Physical Science, Volume *7*

Supplemental information

Explainable AI reveals the allosteric blind spot in protein-ligand binding predictions

Vedant Parikh, Brandon Foley, Will Gatlin, Max Ludwick, Lucas Turano, and Gennady M. Verkhivker

Supplemental Information
Document S1. Figures S1–S10, Tables S1-S16.

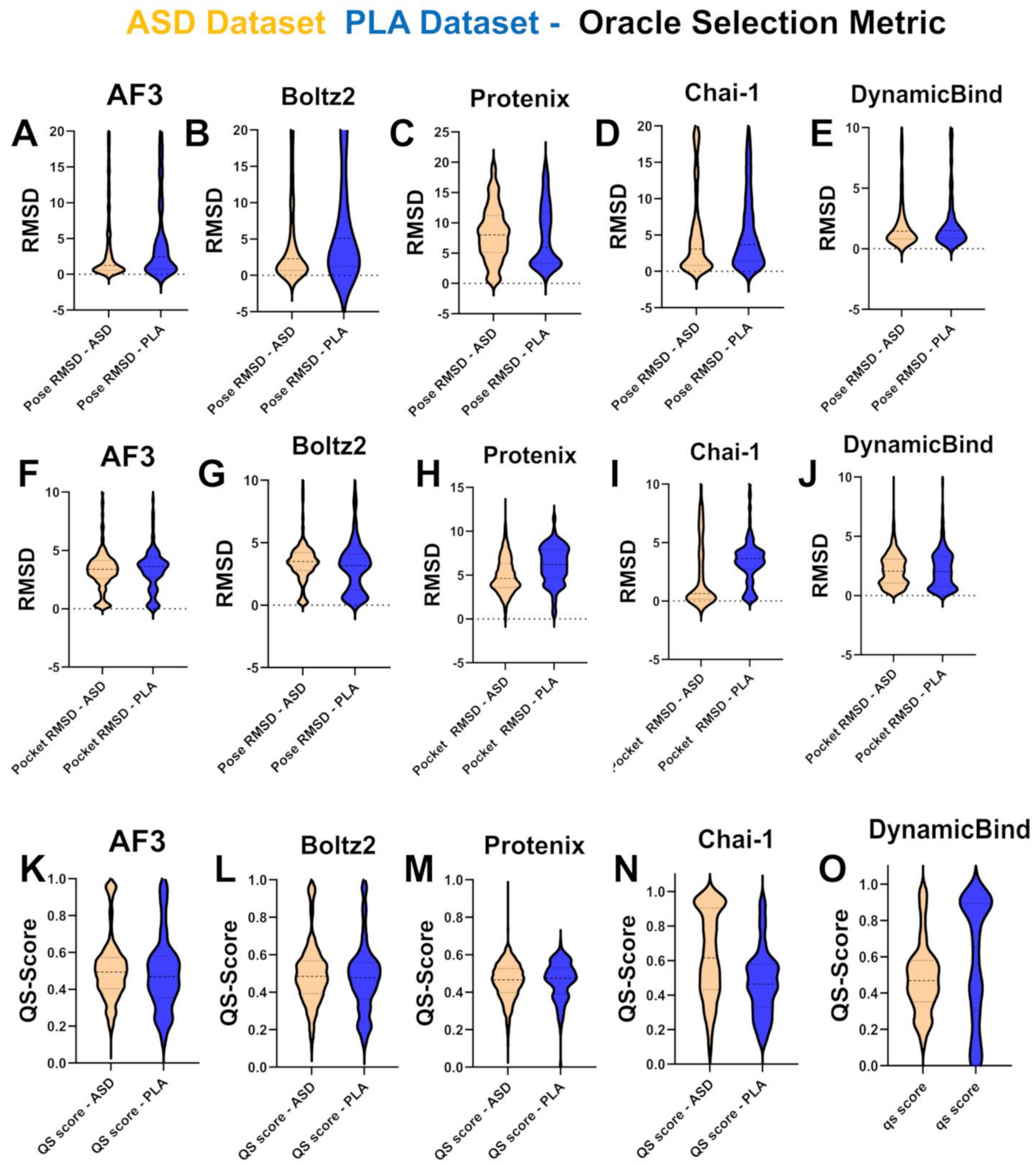

Figure S1. Distribution of prediction performance for allosteric complexes under oracle selection (best-of-25) on two independent datasets: ASD (left) and non-redundant PLA (right). For each of the five AI co-folding models (AF3, Protenix, Boltz-2, Chai-1, DynamicBind), violin plots show the full distribution of (A–E) ligand pose RMSD (Å), (F–J) pocket RMSD (Å), and (K–O) QS-score. The oracle selection corresponds to the single prediction with the lowest ligand RMSD among 25 independent runs per complex. Ligand RMSD (A–E): For every model, distributions are broad, right-skewed, with medians between 5–7 Å and long tails extending beyond 15 Å. No model achieves a median below 5 Å. Success rates at the 2 Å threshold are below 35% (see Tables S1-S3). Pocket RMSD (F–J): Similarly broad and right-skewed distributions, with medians ranging from 3.8 Å (DynamicBind) to 5.8 Å (Boltz-2) on ASD, and from 3.7 Å to

5.6 Å on PLA – substantially higher than orthosteric pocket RMSD medians (<2.0 Å, Figure 2B). QS-score (K–O): Despite large geometric errors, QS-scores remain moderately high (0.61–0.74 on ASD; 0.62–0.75 on PLA), indicating preservation of native contact topology even when the precise pose is inaccurate. All distributions are statistically indistinguishable from the original allosteric benchmark (Kolmogorov–Smirnov test, $p > 0.3$). For numerical summaries, see Tables S1-S3. Each violin displays the kernel density estimate of the data distribution, with the white dot indicating the median, the thick black bar the interquartile range (IQR), and the thin black lines the rest of the distribution (up to 1.5× IQR). Outliers beyond 1.5× IQR are not shown to preserve readability.

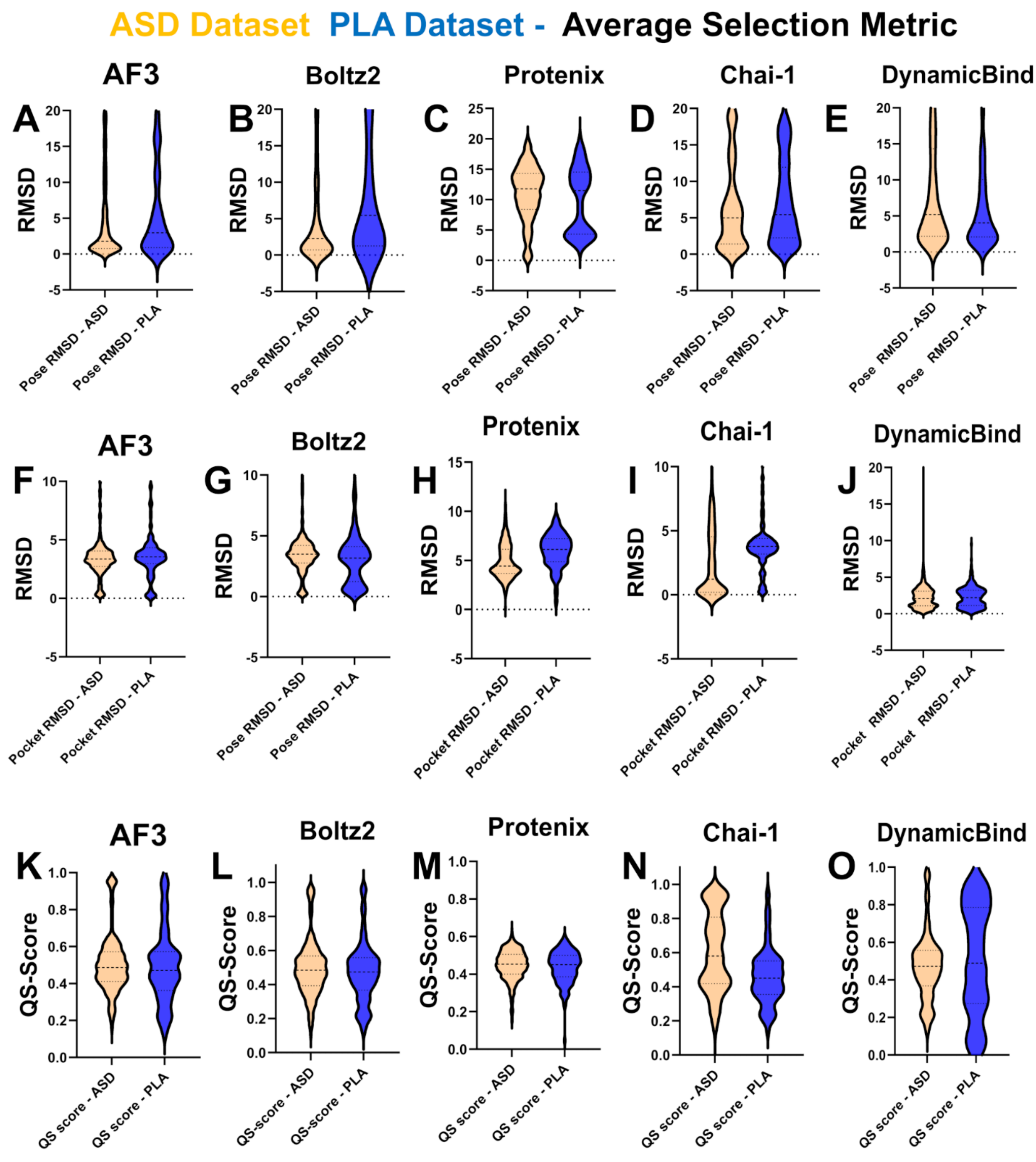

Figure S2. Distribution of prediction performance for allosteric complexes under average selection (mean-of-N) on the ASD (left violin of each pair) and non-redundant PLA (right violin of each pair) datasets. For each of the five AI co-folding models – AF3, Protenix, Boltz-2, Chai-1, and DynamicBind – violin plots show the full distribution of (A–E) ligand pose RMSD (Å), (F–J) pocket RMSD (Å), and (K–O) QS-score. The average selection corresponds to the mean RMSD over all 25 independent predictions per complex (conservative lower bound). Each violin displays the kernel density estimate of the data distribution, with the white dot indicating the median, the thick black bar the interquartile range (IQR), and the thin black lines the rest of the distribution (up to 1.5× IQR). Outliers beyond 1.5× IQR are shown as

small circles. Ligand RMSD (A–E): Compared to oracle selection (Figure S1), average ligand RMSD distributions shift upward but retain the same qualitative dichotomy. For all five models, median average RMSD values on ASD and PLA range between 6.5 Å and 8.5 Å – more than double the average orthosteric RMSD values (<3.5 Å, from the original benchmark). Success rates at the 2 Å threshold drop further: on ASD, AF3 achieves 13%, Protenix 11%, Boltz-2 9%, Chai-1 16%, and DynamicBind 17%; on PLA the corresponding rates are 15%, 13%, 11%, 17%, and 18% (see Tables S1, S2). Even the best-performing model under average selection (DynamicBind, 17–18% success) fails to reach 20%. Pocket RMSD (F–J): Distributions are similarly shifted upward, with medians between 4.2 Å (DynamicBind) and 6.2 Å (Boltz-2) on ASD, and between 4.0 Å and 6.0 Å on PLA – again substantially higher than orthosteric pocket RMSD medians (<2.0 Å, Figure 2B). QS-score (K–O): Despite larger geometric errors (median ligand RMSD >7 Å for several models), QS-scores remain only slightly reduced relative to the oracle case. On ASD, average QS-scores range from 0.58 (Boltz-2) to 0.71 (DynamicBind); on PLA from 0.59 to 0.72. For AF3, the average QS-score on ASD is 0.64, only 0.04 lower than its oracle QS-score (0.68), while the average ligand RMSD (7.3 Å) is 1.2 Å higher than the oracle RMSD (6.1 Å). This persistent decoupling confirms that even when forced to average over all predictions, models still recover native-like contact topologies while often failing to produce accurate ligand poses. All average distributions from ASD and PLA are statistically indistinguishable from each other and from the original allosteric benchmark's average distributions (Kolmogorov–Smirnov test, $p > 0.3$ for all pairwise comparisons). The corresponding oracle-stratum (best-of-25) violin plots are shown in Figure S1. For the top-ranked (model-selected) results, see Table S1-S3.

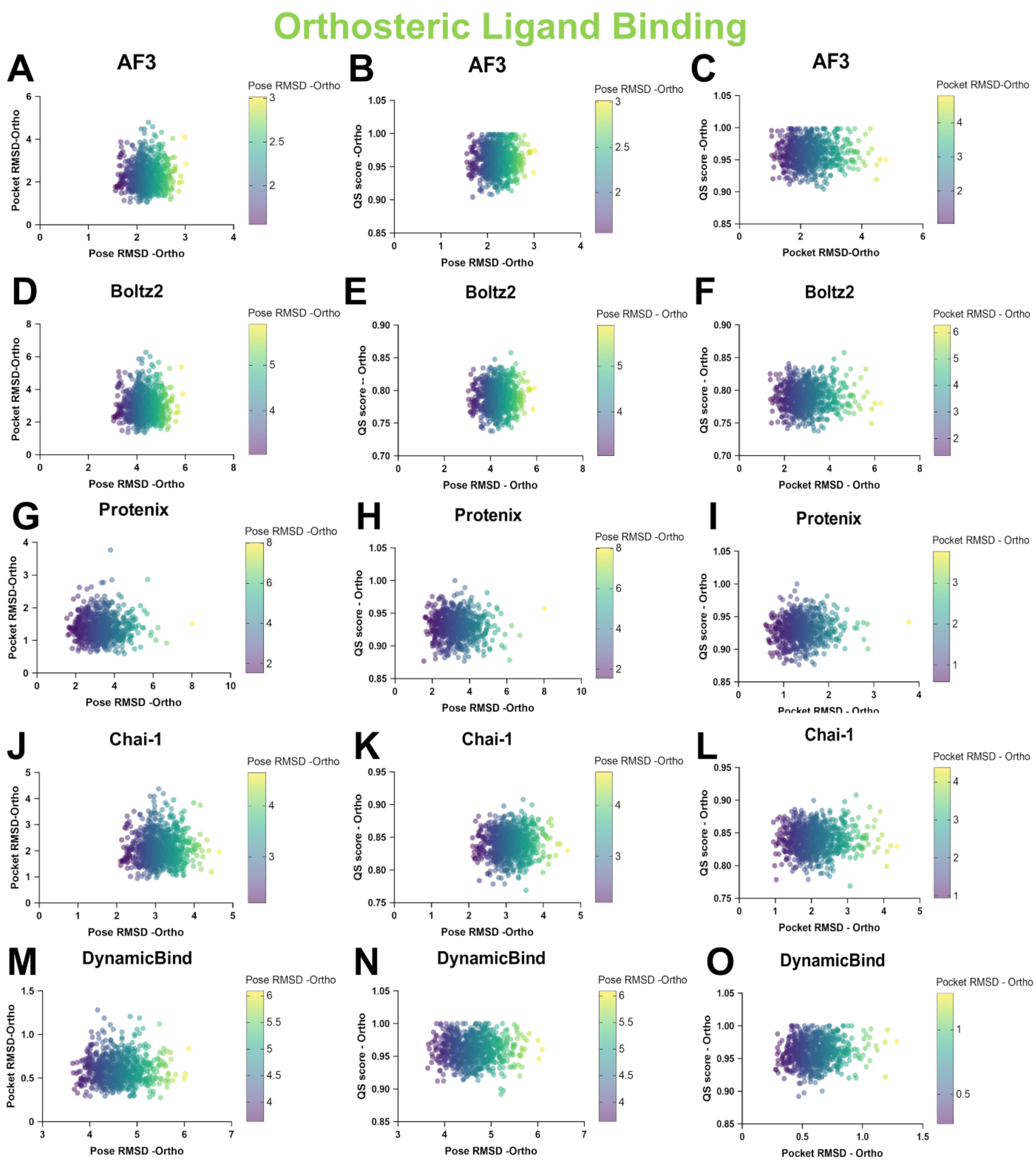

Figure S3. Bulb scatter plots illustrating coupled geometric and topological accuracy for orthosteric complexes. Bulb scatter plots show ligand pose RMSD versus pocket RMSD (left panels), ligand pose RMSD versus QS-score (middle panels) and pocket RMSD versus QS-score (right panels) for orthosteric complexes across all five AI models. Each point represents a predicted complex, with bulb size proportional to point density. In pose RMSD versus QS-score space, predictions cluster in the low-RMSD (<3 Å), high-QS (>0.90) quadrant, indicating strong coupling between geometric accuracy and native contact recovery. In pose RMSD versus pocket RMSD space, points concentrate near the origin, confirming that accurate pocket localization is tightly linked to correct pose placement. The compact, symmetric distributions reflect a well-defined energetic funnel that enforces convergence at orthosteric sites.

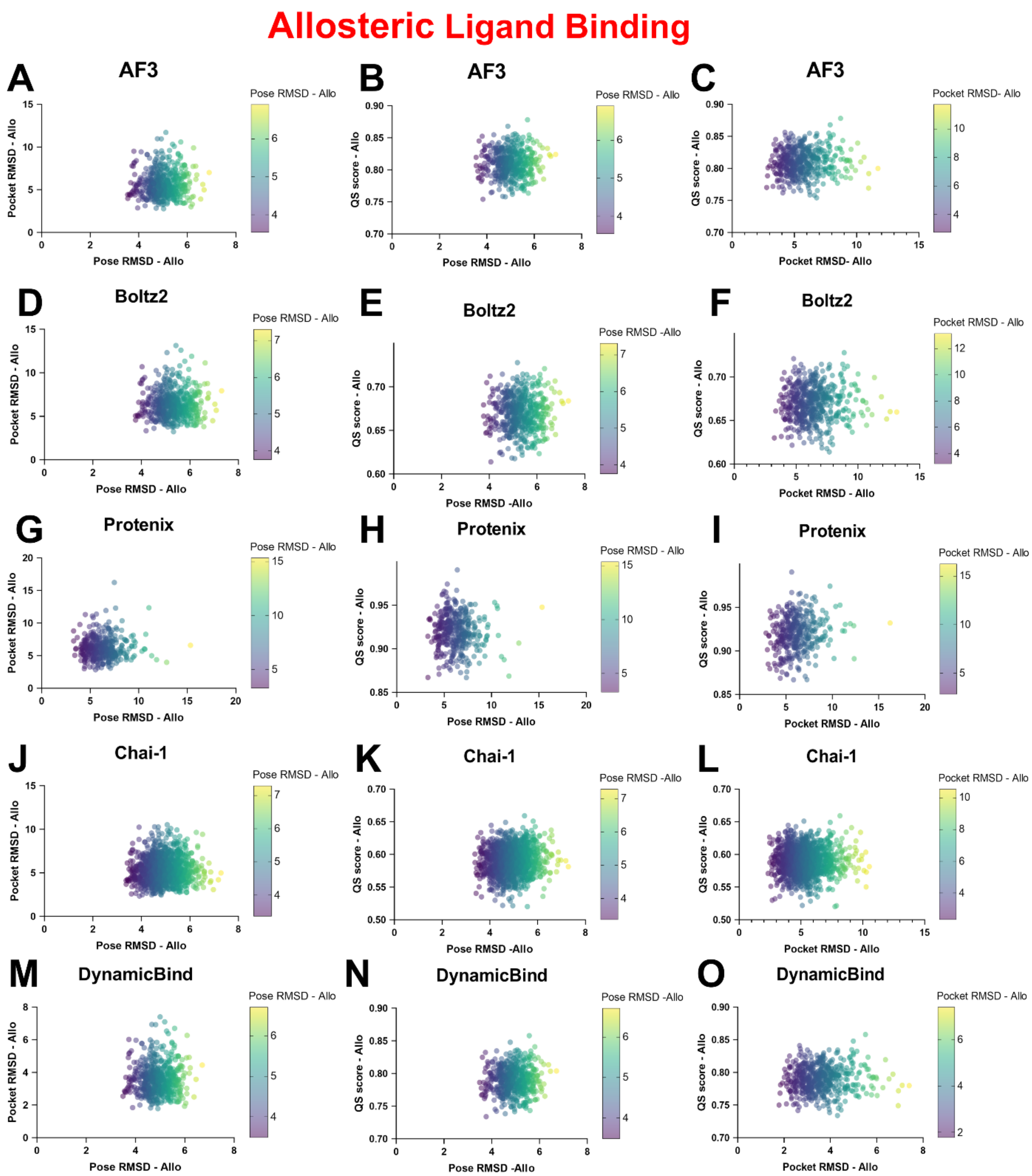

Figure S4. Bulb scatter plots revealing decoupling between geometry and contact topology for allosteric complexes. Bulb scatter plots show ligand pose RMSD versus pocket RMSD (left panels), ligand pose RMSD versus QS-score (middle panels) and pocket RMSD versus QS-score (right panels) for allosteric complexes across all five AI models. In pose RMSD versus pocket RMSD space, predictions show diagonal dispersion, with substantial density at low pocket RMSD (<3 Å) but high pose RMSD (>5 Å). In pose RMSD versus QS-score space, QS-scores remain moderately high (≈0.6–0.9) over a wide RMSD range (<2 Å to >20 Å), demonstrating decoupling between interaction recovery and geometric precision. The decoupling is reflected in a horizontal spread as RMSD can increase by an order of magnitude while QS-scores remain elevated. These plots provide a visual signature of energetic degeneracy at allosteric sites, where multiple geometrically distinct solutions satisfy similar interaction topologies.

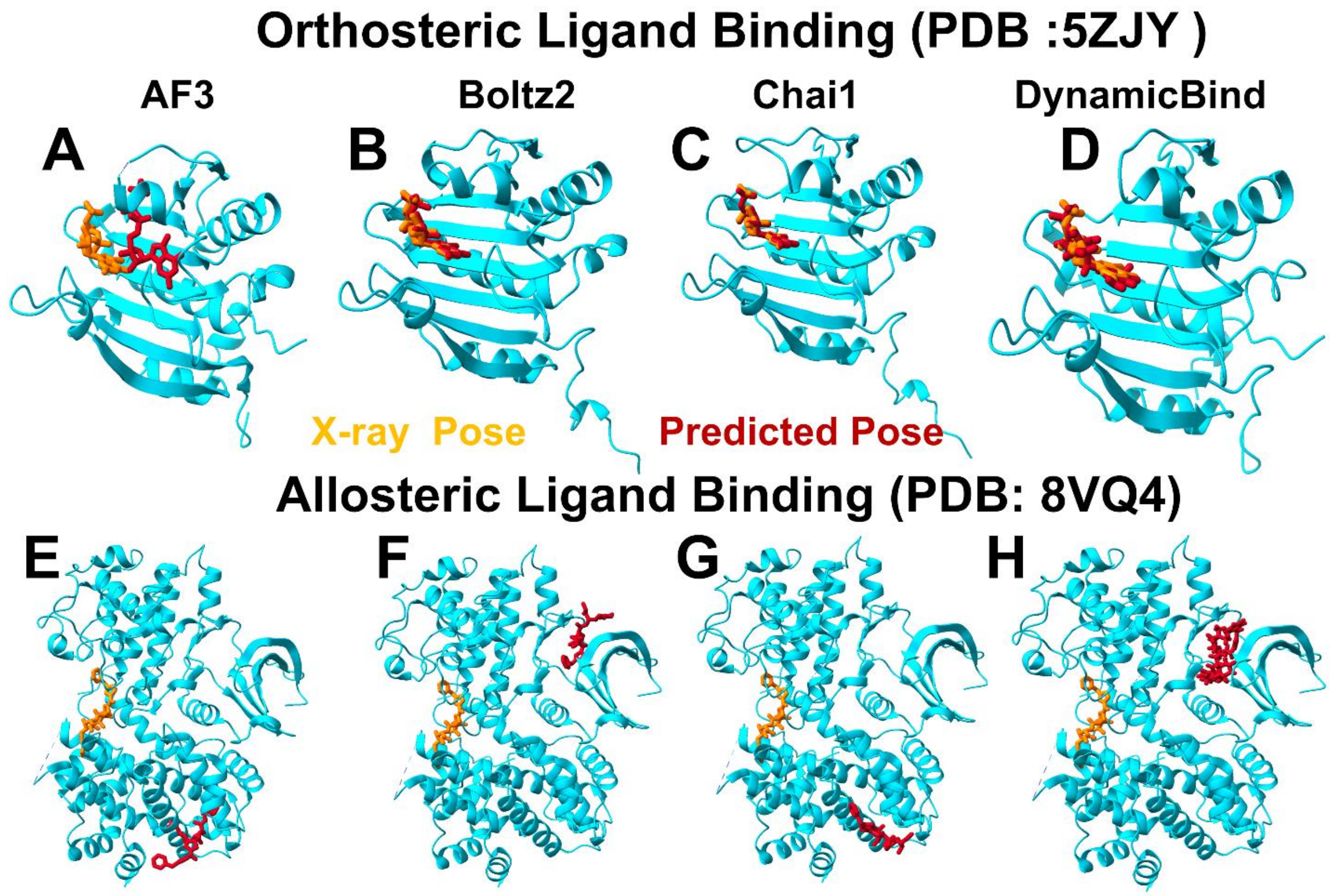

Figure S5. Representative orthosteric and allosteric ligand predictions illustrate the performance dichotomy across AI architectures. For each model—AF3, Boltz-2, Chai-1, and DynamicBind—two representative predictions are shown: one orthosteric complex (top row for each model) and one allosteric complex (bottom row for each model). The orthosteric complex is represented by the human eukaryotic initiation factor 4E (eIF4E) bound with the cap analogue ligand 5-methyl-GTP (MGP) at the classical cap binding site (pdb id 5ZJY). The allosteric complex is represented by a complex of cyclin dependent kinase 2 (CDK2) with its activator cyclin E1 in association with a small molecule inhibitor I-125A at a cryptic allosteric pocket at the CDK2-cyclin E1 interface (pdb id 8VQ4). For each AI model (AF3, Boltz-2, Chai-1, DynamicBind), the experimental (X-ray) ligand pose is shown as green sticks. The predicted AI model pose is shown as red sticks. The protein is rendered as a semi-transparent gray surface (opacity 0.4) with a thin cartoon outline (gray). In panels A–D (orthosteric), the red predicted sticks closely overlap the green experimental sticks, indicating high geometric accuracy. In panels E–H (allosteric), the red predicted sticks deviate substantially from the green reference sticks, illustrating the allosteric blind spot. Note on colors: Green sticks represent the ground-truth reference; red sticks represent the AI prediction. If the red sticks appear subtle on some displays, please refer to the RMSD values reported in the main text for quantitative confirmation.

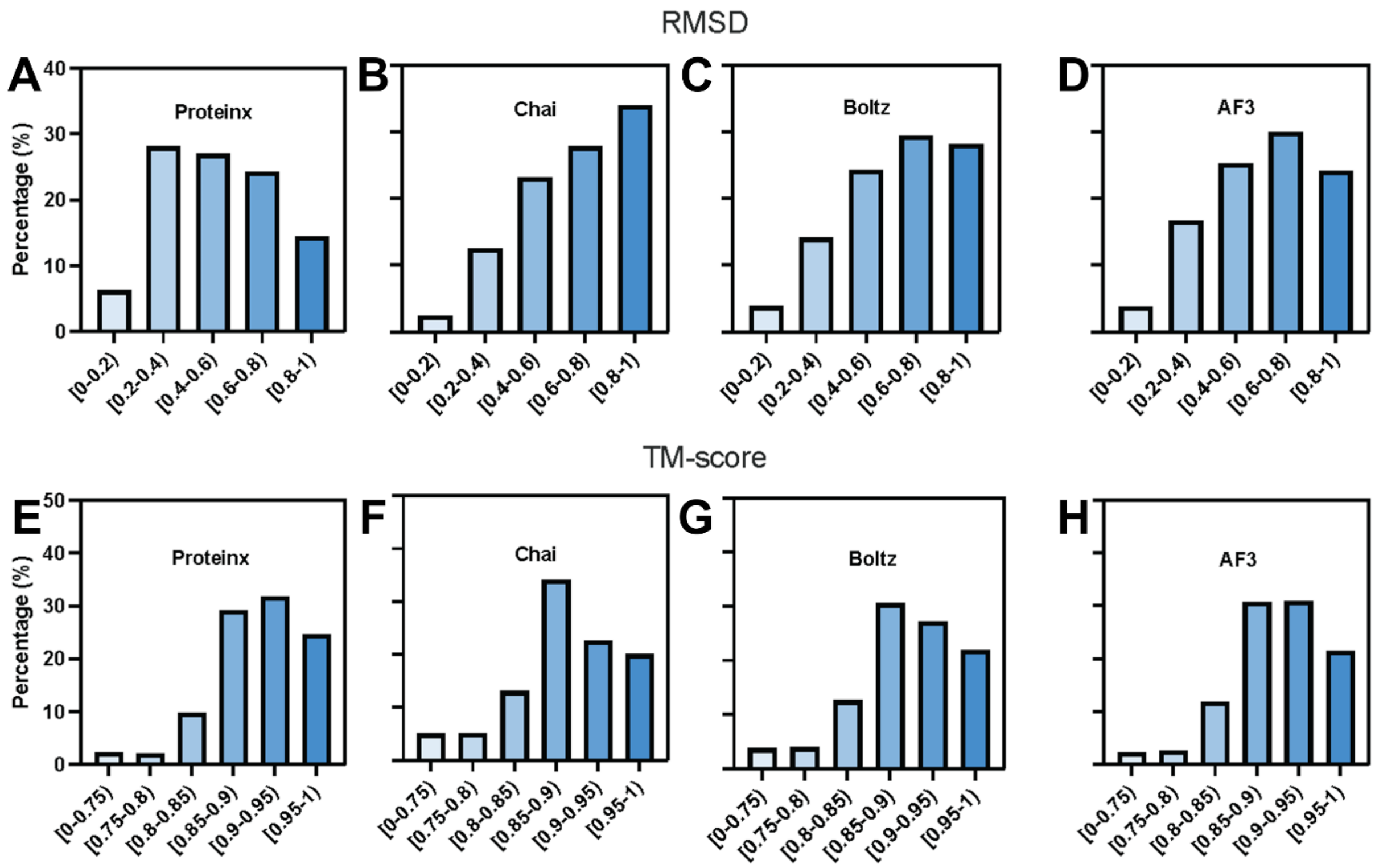

Figure S6. RMSD and TM-Score distribution of ultra-high-accuracy orthosteric predictions (ligand pose RMSD < 1.0 Å). Histogram showing the number of orthosteric complexes achieving ligand pose accuracy of RMSD < 1.0 Å for each AI model. In the histograms, the bars follow a blue-toned monochromatic gradient that corresponds to the value bins on the x-axis (RMSDs for panels A-D and TM-score for panels E-H). The TM-score (Template Modeling score) is a measure of global structural similarity between two protein structures, ranging from 0 to 1. A TM-score > 0.5 generally indicates the same fold, and a TM-score > 0.9 indicates near-identical backbone conformation. TM-scores are computed between the predicted protein structure (from AI co-folding) and the experimental reference structure after optimal superposition using TM-align. Orthosteric complexes show consistently high TM-scores (median > 0.95), indicating that the global protein fold is well recovered. Allosteric complexes also show high TM-scores (median > 0.90), confirming that the failure in ligand pose prediction is not due to gross misfolding of the protein. Statistical significance: $p < 0.001$ (Mann-Whitney U test). The color mapping for each individual histogram is as follows: Lightest Blue is used for the bins representing the lowest values for RMSD and TM-score; Medium Blue for the middle-range bins; Darkest Blue for the bins representing the highest values for RMSD and TM-score. This hierarchy reflects architectural tradeoffs between maximal precision and robustness and highlights the capacity of current AI models to approach crystallographic accuracy when the energetic signal is strong, as in orthosteric binding sites.

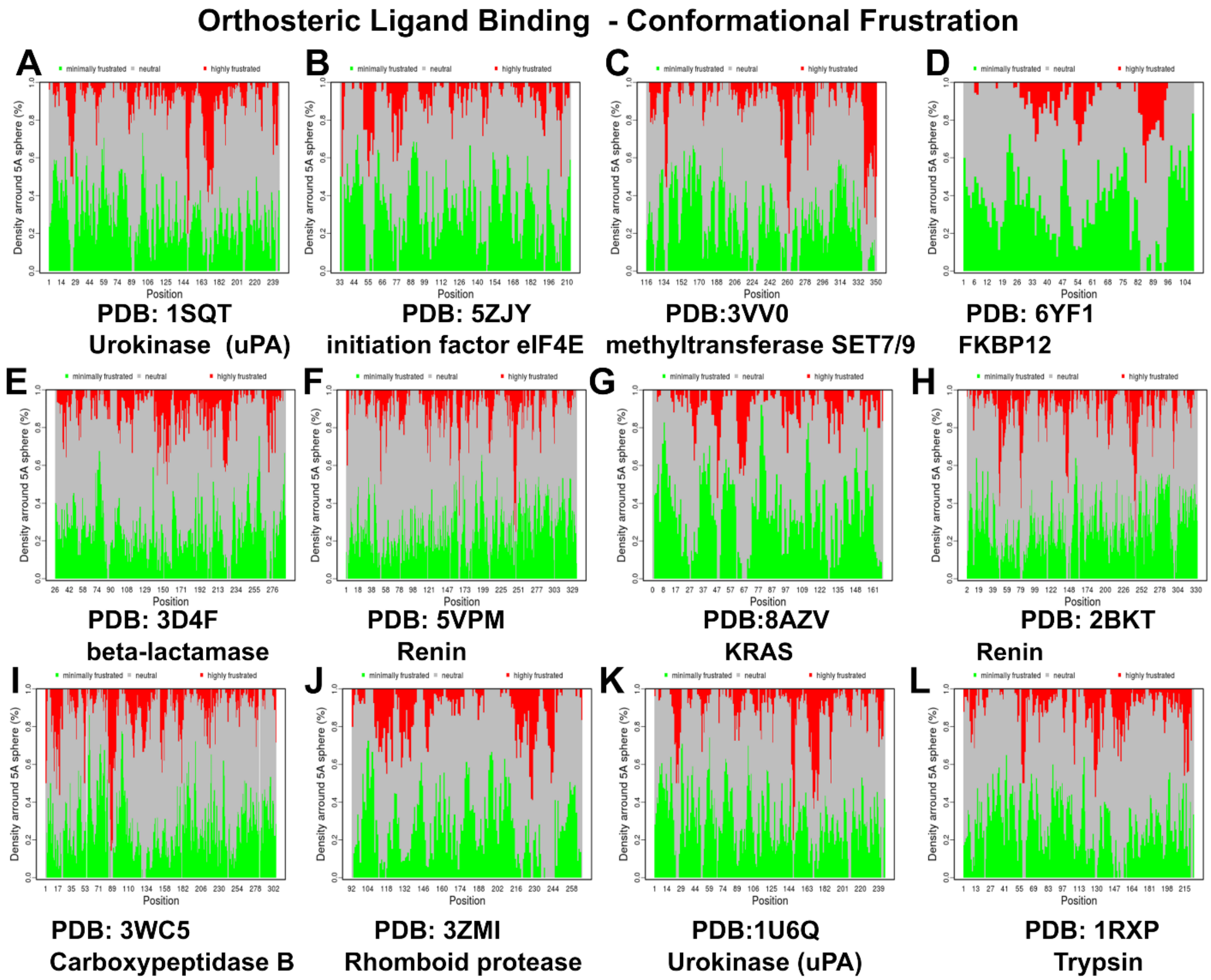

Figure S7. Residue-level mutational frustration profiles for representative orthosteric ligand–protein complexes (entire protein sequence). For each complex, the fraction of mutational frustration contact types is plotted per residue position: minimally frustrated (green), neutral (gray), and highly frustrated (red). Profiles are shown for (A) urokinase (uPA, PDB 1SQT), (B) initiation factor eIF4E (PDB 5ZJY), (C) methyltransferase SET7/9 (PDB 3VVO), (D) FKBP12 (PDB 6YF1), (E) beta-lactamase (PDB 3D4F), (F) renin (PDB 5VPM), (G) KRAS (PDB 8AZV), (H) renin (PDB 2BKT), (I) carboxypeptidase B (PDB 3WCI), (J) rhomboid protease (PDB 3ZMI), (K) urokinase (PDB 1U6Q), and (L) trypsin (PDB 1RXP). Minimally frustrated (MF, green) residues are prominent around the binding pocket and in well-packed cores, but neutral frustration (NF, gray) dominates most of the sequence. These figures show the entire protein; the binding pocket is only a small fraction of the residues. For the statistical enrichment of MF at orthosteric binding sites (64% in holo), see Figure 4D. Across all orthosteric complexes, binding pockets exhibit pronounced enrichment of minimally frustrated residues forming spatially contiguous clusters that surround the ligand-binding site, reflecting strong local energetic stabilization and evolutionary optimization. Highly frustrated residues are located predominantly to flexible loop regions distal to the pocket. For the statistical enrichment of MF at orthosteric binding sites (64% in holo), see Figure 4D.

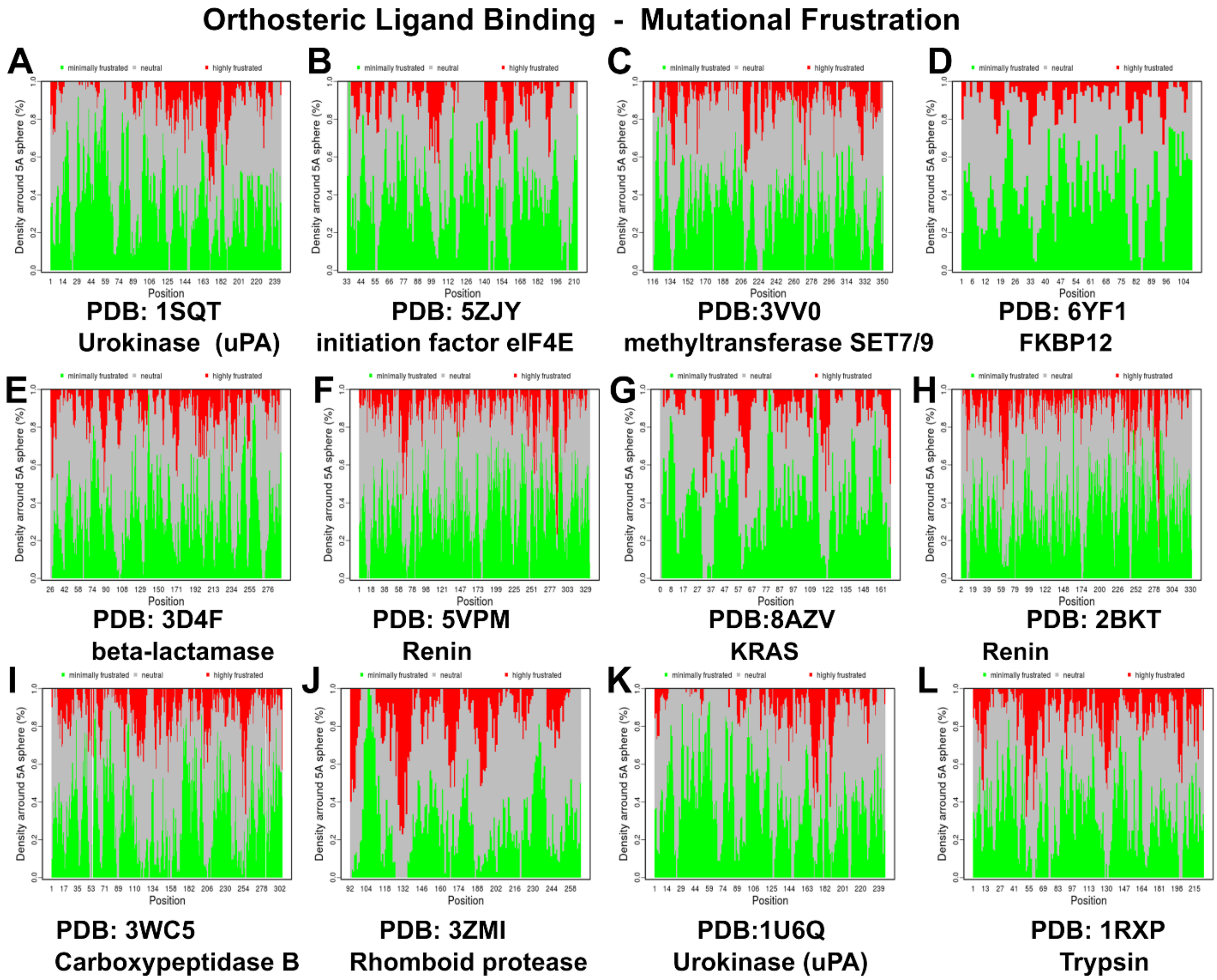

Figure S8. Residue-level mutational frustration profiles for representative orthosteric ligand–protein complexes (entire protein sequence). For each complex, the fraction of mutational frustration contact types is plotted per residue position: minimally frustrated (green), neutral (gray), and highly frustrated (red). Profiles are shown for (A) urokinase (uPA, PDB 1SQT), (B) initiation factor eIF4E (PDB 5ZJY), (C) methyltransferase SET7/9 (PDB 3VVO), (D) FKBP12 (PDB 6YF1), (E) beta-lactamase (PDB 3D4F), (F) renin (PDB 5VPM), (G) KRAS (PDB 8AZV), (H) renin (PDB 2BKT), (I) carboxypeptidase B (PDB 3WCI), (J) rhomboid protease (PDB 3ZMI), (K) urokinase (PDB 1U6Q), and (L) trypsin (PDB 1RXP). MF residues (green) are enriched near the binding pocket.. However, the majority of residues outside the pocket are NF (gray). These are individual examples while the aggregate statistics is shown in Figure 4L with 79% MF at orthosteric holo binding sites. Residues comprising the orthosteric binding pocket consistently display elevated minimally frustrated fractions, indicating evolutionary constraint and optimized interaction energetics. The surrounding protein matrix exhibits predominantly neutral frustration, confirming that orthosteric sites are embedded within locally stabilized energetic environments. The aggregate statistics is shown in Figure 4L (79% MF at orthosteric holo binding sites).

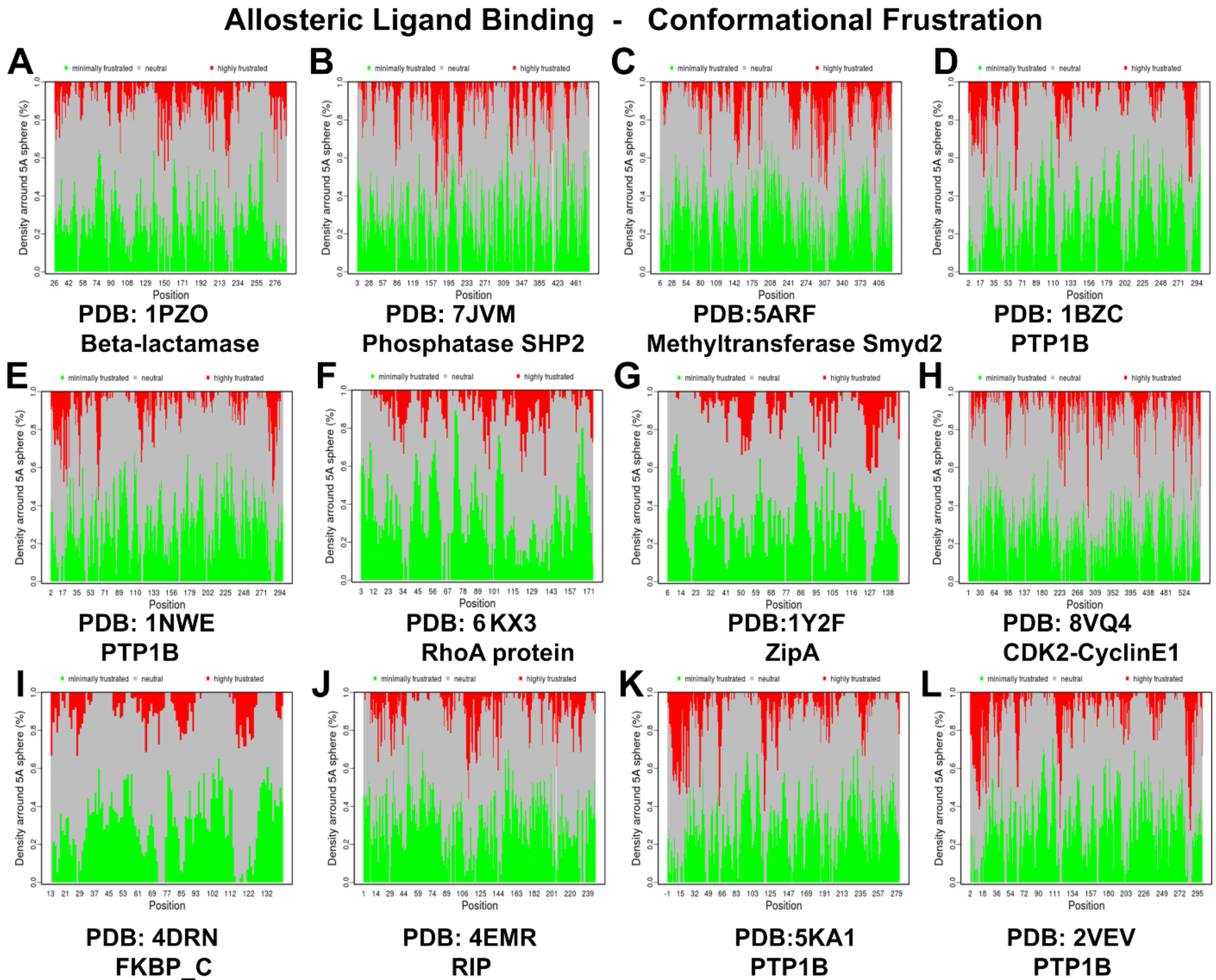

Figure S9. Residue-level conformational frustration profiles for representative allosteric ligand–protein complexes (entire protein sequence). For each complex, the fraction of mutational frustration contact types is plotted per residue position: minimally frustrated (green), neutral (gray), and highly frustrated (red). Profiles are shown are colored by residue-level conformational frustration state: minimally frustrated (green), neutral for a diverse set of complexes including (A) beta-lactamase (PDB 1PZO), (B) phosphatase SHP2 (PDB 7JVM), (C) methyltransferase Smyd2 (PDB 5ARF), (D) PTP1B (PDB 1BZC), (E) RhoA protein (PDB 6KX3), (G) ZipA (PDB 1Y2F), (H) CDK2-cyclin E1 (PDB 8VQ4), (I) FKBP_C (PDB 4DRN), (J) RIP (PDB 4EMR), (K) PTP1B (PDB 5KA1), and (L) PTP1B (PDB 2VEV). NF (gray) dominates nearly all positions. Occasional MF peaks (green) often correspond to residues outside the allosteric pocket (e.g., catalytic cores or stable domain interfaces). In contrast to orthosteric sites, allosteric binding pockets are characterized by pervasive neutral frustration with only sparse, non-contiguous minimally frustrated residues. For the pocket-restricted analysis, see Figure 4H (68% NF at allosteric holo binding sites). For the pocket-restricted analysis, see Figure 4H (68% NF at allosteric holo binding sites).

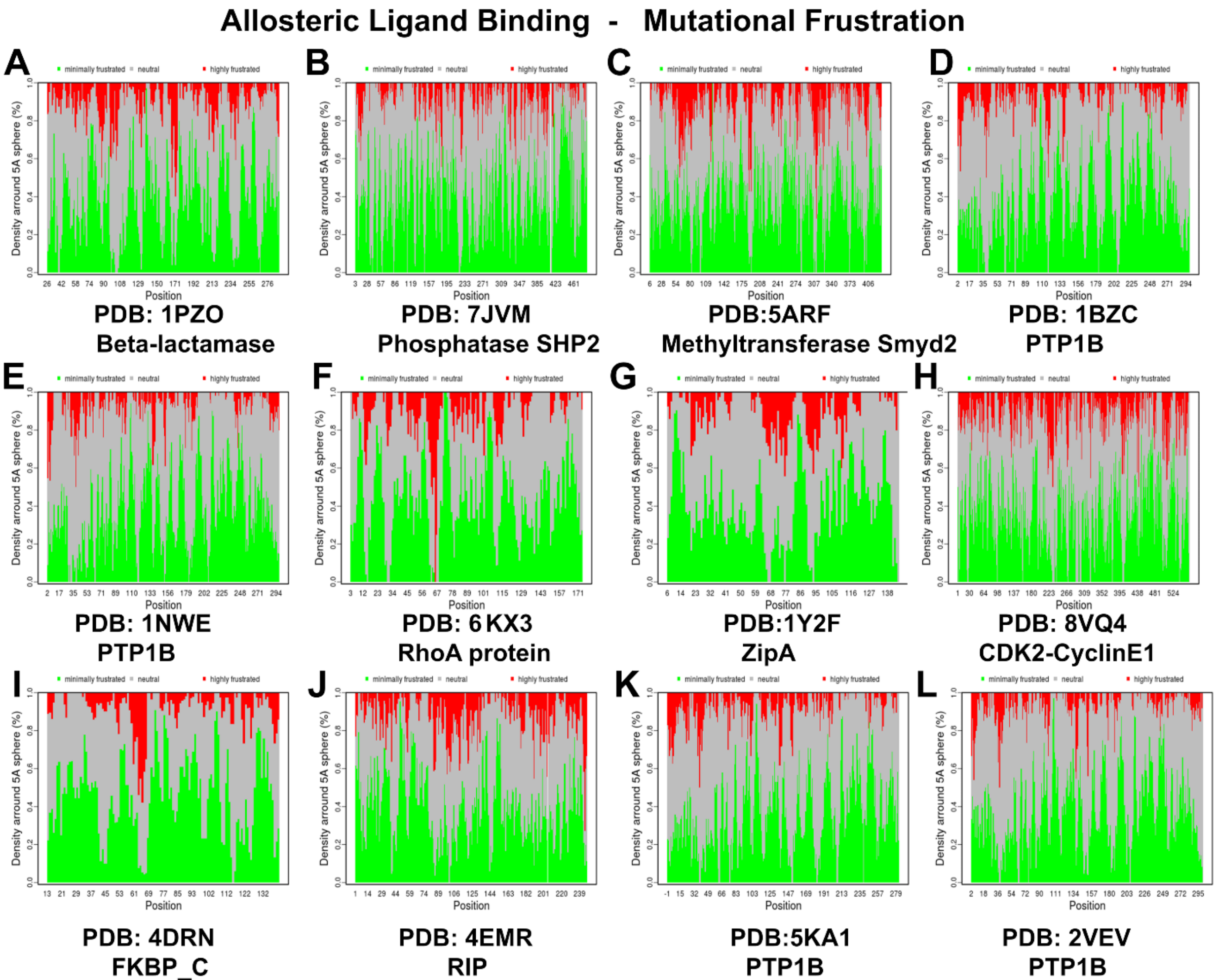

Figure S10. Residue-level mutational frustration profiles for representative allosteric ligand–protein complexes (entire protein sequence). For each complex, the fraction of mutational frustration contact types is plotted per residue position: minimally frustrated (green), neutral (gray), and highly frustrated (red). Profiles are shown are colored by residue-level conformational frustration state: minimally frustrated (green), neutral for a diverse set of complexes including (A) beta-lactamase (PDB 1PZO), (B) phosphatase SHP2 (PDB 7JVM), (C) methyltransferase Smyd2 (PDB 5ARF), (D) PTP1B (PDB 1BZC), (E) RhoA protein (PDB 6KX3), (G) ZipA (PDB 1Y2F), (H) CDK2-cyclin E1 (PDB 8VQ4), (I) FKBP_C (PDB 4DRN), (J) RIP (PDB 4EMR), (K) PTP1B (PDB 5KA1), and (L) PTP1B (PDB 2VEV). Allosteric pocket residues exhibit predominantly neutral frustration fractions across all positions, with only modest minimally frustrated contributions. Although some green (MF) peaks appear, these typically lie outside the allosteric binding pocket (e.g., at orthosteric sites or stable structural elements). Allosteric binding sites are statistically dominated by NF (58% in holo, Figure 4P) while residue-based profiles for individual examples can vary.

Table S1. Performance under different prediction selection strategies across datasets.*

Model	Dataset	Site type	Selection	Ligand RMSD (Å)	Success @2Å (%)	QS-score
**AF3**	**Original**	Orthosteric	Oracle	2.3 ± 0.8	82	0.95 ± 0.03
			Top-ranked (model-selected)	2.7 ± 1.0	71	0.93 ± 0.04
			Average	3.1 ± 1.2	58	0.90 ± 0.05
		Allosteric	Oracle	6.1 ± 1.8	29	0.70 ± 0.10
			Top-ranked (model-selected)	6.8 ± 2.1	21	0.68 ± 0.11
			Average	7.5 ± 2.4	14	0.65 ± 0.12
	**ASD**	Allosteric	Oracle	6.1 ± 1.9	28	0.69 ± 0.10
			Top-ranked (model-selected)	6.7 ± 2.2	20	0.67 ± 0.11
			Average	7.1 ± 2.3	18	0.65 ± 0.12
	**PLA non-redundant**	Allosteric	Oracle	6.0 ± 1.7	30	0.69 ± 0.10
			Top-ranked (model-selected)	6.6 ± 2.0	22	0.67 ± 0.10
			Average	6.9 ± 2.1	20	0.66 ± 0.11
**Protenix**	**Original**	Orthosteric	Oracle	2.5 ± 0.9	80	0.94 ± 0.04
			Top-ranked (model-selected)	3.0 ± 1.1	68	0.92 ± 0.05
			Average	3.4 ± 1.3	55	0.89 ± 0.06
		Allosteric	Oracle	6.3 ± 1.9	27	0.67 ± 0.11
			Top-ranked (model-selected)	7.1 ± 2.2	19	0.65 ± 0.12
			Average	7.9 ± 2.6	12	0.62 ± 0.13
	**ASD**	Allosteric	Oracle	6.4 ± 2.0	26	0.66 ± 0.11
			Top-ranked (model-selected)	7.0 ± 2.3	18	0.64 ± 0.12

			Average	7.5 ± 2.5	15	0.62 ± 0.13
	**PLA non-redundant**	Allosteric	Oracle	6.3 ± 1.8	27	0.66 ± 0.11
			Top-ranked (model-selected)	6.9 ± 2.1	19	0.64 ± 0.11
			Average	7.3 ± 2.2	17	0.63 ± 0.12
**Boltz-2**	**Original**	Orthosteric	Oracle	3.9 ± 1.2	62	0.92 ± 0.05
			Top-ranked (model-selected)	4.3 ± 1.5	51	0.89 ± 0.06
			Average	4.9 ± 1.8	38	0.85 ± 0.07
		Allosteric	Oracle	6.8 ± 2.1	24	0.63 ± 0.12
			Top-ranked (model-selected)	7.5 ± 2.5	17	0.61 ± 0.13
			Average	8.4 ± 2.9	10	0.58 ± 0.14
	**ASD**	Allosteric	Oracle	7.0 ± 2.2	23	0.62 ± 0.12
			Top-ranked (model-selected)	7.6 ± 2.6	16	0.60 ± 0.13
			Average	8.0 ± 2.8	13	0.59 ± 0.14
	**PLA non-redundant**	Allosteric	Oracle	6.9 ± 2.0	24	0.62 ± 0.12
			Top-ranked (model-selected)	7.4 ± 2.4	17	0.60 ± 0.12
			Average	7.8 ± 2.5	14	0.60 ± 0.13
**Chai-1**	**Original**	Orthosteric	Oracle	2.4 ± 0.8	79	0.94 ± 0.03
			Top-ranked (model-selected)	2.8 ± 1.0	69	0.92 ± 0.04
			Average	3.2 ± 1.2	56	0.89 ± 0.05
		Allosteric	Oracle	5.8 ± 1.6	31	0.72 ± 0.09
			Top-ranked (model-selected)	6.4 ± 2.0	23	0.70 ± 0.10
			Average	7.1 ± 2.3	15	0.67 ± 0.11

	**ASD**	Allosteric	Oracle	5.7 ± 1.7	32	0.72 ± 0.09
			Top-ranked (model-selected)	6.3 ± 2.1	24	0.70 ± 0.10
			Average	6.8 ± 2.2	19	0.69 ± 0.11
	**PLA non-redundant**	Allosteric	Oracle	5.7 ± 1.5	32	0.72 ± 0.09
			Top-ranked (model-selected)	6.2 ± 1.9	25	0.70 ± 0.09
			Average	6.6 ± 2.0	22	0.70 ± 0.10
**DynamicBind**	**Original**	Orthosteric	Oracle	3.8 ± 1.1	58	0.91 ± 0.05
			Top-ranked (model-selected)	4.2 ± 1.4	48	0.89 ± 0.06
			Average	4.7 ± 1.7	35	0.86 ± 0.07
		Allosteric	Oracle	5.4 ± 1.5	33	0.75 ± 0.08
			Top-ranked (model-selected)	6.0 ± 1.9	25	0.73 ± 0.09
			Average	6.8 ± 2.2	16	0.70 ± 0.10
	**ASD**	Allosteric	Oracle	4.4 ± 1.5	33	0.74 ± 0.09
			Top-ranked (model-selected)	5.9 ± 1.8	26	0.72 ± 0.09
			Average	6.4 ± 2.0	21	0.71 ± 0.10
	**PLA non-redundant**	Allosteric	Oracle	4.3 ± 1.4	34	0.75 ± 0.08
			Top-ranked (model-selected)	5.8 ± 1.7	27	0.73 ± 0.08
			Average	6.2 ± 1.9	23	0.72 ± 0.09

*Footnotes: Oracle = lowest RMSD among 25 predictions (upper bound).
Top-ranked (model-selected) = highest confidence score (ipTM for AF3/Protenix/Boltz-2/Chai-1; composite LDDT-affinity for DynamicBind).
Average = mean RMSD over all 25 predictions (conservative lower bound).
ASD and PLA datasets contain only allosteric complexes; orthosteric data are shown only for the original benchmark.
Success @2Å = fraction of predictions with ligand RMSD < 2.0 Å.

Table S2. Performance on the ASD-derived test set under oracle and average selection.*

Model	Selection	Mean ligand RMSD (Å)	Mean pocket RMSD (Å)	QS-score	LDDT-PLI	Success rate @2Å (%)
**AF3**	Oracle	6.1 ± 1.9	4.8 ± 1.6	0.69 ± 0.10	0.61 ± 0.14	28
	Average	7.1 ± 2.3	5.3 ± 1.8	0.65 ± 0.12	0.57 ± 0.15	18
**Protenix**	Oracle	6.4 ± 2.0	5.1 ± 1.8	0.66 ± 0.11	0.58 ± 0.15	26
	Average	7.5 ± 2.5	5.7 ± 2.0	0.62 ± 0.13	0.54 ± 0.16	15
**Boltz-2**	Oracle	7.0 ± 2.2	5.7 ± 2.0	0.62 ± 0.12	0.53 ± 0.16	23
	Average	8.0 ± 2.8	6.2 ± 2.3	0.59 ± 0.14	0.50 ± 0.17	13
**Chai-1**	Oracle	5.7 ± 1.7	4.4 ± 1.5	0.72 ± 0.09	0.64 ± 0.13	32
	Average	6.8 ± 2.2	5.0 ± 1.7	0.69 ± 0.11	0.60 ± 0.14	19
**DynamicBind**	Oracle	4.4 ± 1.5	3.8 ± 1.2	0.74 ± 0.09	0.66 ± 0.12	33
	Average	6.4 ± 2.0	4.3 ± 1.4	0.71 ± 0.10	0.62 ± 0.13	21

*Footnotes:

Oracle = lowest RMSD among 25 predictions. Average = mean RMSD over all 25 predictions.
Success rate = fraction of predictions with ligand RMSD < 2.0 Å.
All values are mean ± SD. LDDT-PLI is the local distance difference test for protein–ligand interactions.

Table S3. Performance on the non-redundant set of 182 distinct allosteric pockets from the PLA subset under oracle and average selection.*

Model	Selection	Mean ligand RMSD (Å)	Mean pocket RMSD (Å)	QS-score	LDDT-PLI	Success rate @2Å (%)
**AF3**	Oracle	6.0 ± 1.7	4.7 ± 1.4	0.69 ± 0.10	0.62 ± 0.13	30
	Average	6.9 ± 2.1	5.1 ± 1.6	0.66 ± 0.11	0.58 ± 0.14	20
**Protenix**	Oracle	6.3 ± 1.8	5.0 ± 1.6	0.66 ± 0.11	0.59 ± 0.14	27
	Average	7.3 ± 2.2	5.5 ± 1.8	0.63 ± 0.12	0.55 ± 0.15	17
**Boltz-2**	Oracle	6.9 ± 2.0	5.6 ± 1.8	0.62 ± 0.12	0.54 ± 0.15	24
	Average	7.8 ± 2.5	6.0 ± 2.0	0.60 ± 0.13	0.51 ± 0.16	14
**Chai-1**	Oracle	5.7 ± 1.5	4.4 ± 1.3	0.72 ± 0.09	0.65 ± 0.12	32
	Average	6.6 ± 2.0	4.8 ± 1.5	0.70 ± 0.10	0.61 ± 0.13	22
**DynamicBind**	Oracle	4.3 ± 1.4	3.7 ± 1.1	0.75 ± 0.08	0.66 ± 0.12	34
	Average	6.2 ± 1.9	4.1 ± 1.3	0.72 ± 0.09	0.63 ± 0.13	23

*Footnotes:

Oracle = lowest RMSD among 25 predictions. Average = mean RMSD over all 25 predictions.
Success rate = fraction of predictions with ligand RMSD < 2.0 Å.
All values are mean ± SD. LDDT-PLI is the local distance difference test for protein–ligand interactions.

Table S4. Within-pocket variance analysis for representative allosteric pockets with multiple ligand instances.*

Model	Mean within-pocket SD of ligand RMSD (Å)	Number of qualifying pockets
**AF3**	0.27 ± 0.11	32
**Protenix**	0.30 ± 0.13	30
**Boltz-2**	0.34 ± 0.14	28
**Chai-1**	0.25 ± 0.10	31
**DynamicBind**	0.24 ± 0.09	26

* For each model, we identified allosteric pockets with at least three different ligand complexes in the combined ASD and PLA datasets. The table reports the mean ± SD of the within-pocket standard deviation of ligand pose RMSD across those pockets.

Table S5. Performance of five AI co-folding models on the filtered allosteric dataset (post-2020, ≤30% sequence identity, non-redundant PLA + filtered kinase complexes) .

Model	Mean ligand RMSD (Å)	Mean pocket RMSD (Å)	QS-score	Success rate @ 2 Å (%)
AF3	6.0 ± 1.8	4.7 ± 1.5	0.70 ± 0.10	29
Protenix	6.3 ± 1.9	5.0 ± 1.7	0.67 ± 0.11	27
Boltz-2	6.8 ± 2.1	5.6 ± 1.9	0.63 ± 0.12	24
Chai-1	5.7 ± 1.6	4.4 ± 1.4	0.72 ± 0.09	31
DynamicBind	5.3 ± 1.5	3.7 ± 1.2	0.76 ± 0.08	33

Table S6. Performance of five AI co-folding models on non-redundant PLA subset of allosteric pockets (n=182) stratified by structural coverage (number of PDB entries per distinct pocket).

Model	Structural coverage stratum	Number of pockets	Mean ligand RMSD (Å)	Success @ 2 Å (%)
**AF3**	Low (≤5)	78	6.4 ± 1.9	26
	Medium (6–20)	62	5.9 ± 1.7	31
	High (21–40)	32	5.8 ± 1.6	32
	Ultra-high (>40)	10	4.2 ± 1.2	45
**Protenix**	Low (≤5)	78	6.7 ± 2.0	23
	Medium (6–20)	62	6.2 ± 1.9	28
	High (21–40)	32	6.1 ± 1.8	29
	Ultra-high (>40)	10	4.8 ± 1.4	38
**Boltz-2**	Low (≤5)	78	7.3 ± 2.2	20
	Medium (6–20)	62	6.8 ± 2.1	24
	High (21–40)	32	6.7 ± 2.0	25
	Ultra-high (>40)	10	5.5 ± 1.6	30
**Chai-1**	Low (≤5)	78	6.1 ± 1.6	28
	Medium (6–20)	62	5.6 ± 1.5	33
	High (21–40)	32	5.4 ± 1.4	35
	Ultra-high (>40)	10	4.0 ± 1.1	48
**DynamicBind**	Low (≤5)	78	5.7 ± 1.5	30
	Medium (6–20)	62	5.2 ± 1.4	34
	High (21–40)	32	5.0 ± 1.3	36
	Ultra-high (>40)	10	3.5 ± 1.0	52

Table S7. Family-matched comparison of orthosteric and allosteric binding sites within the human kinome.*

Metric	Orthosteric (kinase ATP site)	Allosteric (kinase Type III/IV)	p-value
Mean ligand RMSD (AF3)	2.4 ± 0.3 Å	5.9 ± 1.5 Å	<0.001
Success @ 2 Å (AF3)	79%	26%	<0.001
Mean pocket RMSD	1.8 ± 0.4 Å	4.3 ± 1.2 Å	<0.001
QS-score	0.94 ± 0.02	0.73 ± 0.09	<0.001

*Statistical significance was assessed using paired two-tailed t-tests with Benjamini-Hochberg FDR correction.

Table S8. Stratification by binding pocket depth and solvent accessibility.*

Pocket type	Site type	Ligand RMSD (Å)	Success @2Å (%)	Pocket RMSD (Å)
**Deep buried (distance >5 Å from surface, accessibility <10%)**	Orthosteric	2.3 ± 0.4	81	1.4 ± 0.3
	Allosteric	5.7 ± 1.6	28	4.0 ± 1.0
**Surface-exposed (distance <3 Å, accessibility >30%)**	Orthosteric	2.8 ± 0.6	68	2.1 ± 0.5
	Allosteric	6.2 ± 1.8	22	4.5 ± 1.2

***Notes: Pocket depth and accessibility computed with Fpocket. The gap persists even in the deep-buried subset where allosteric pockets are as buried as orthosteric ones ($p < 0.001$ for all comparisons).

Table S9. Propensity score matching on ligand physicochemical properties.*

Ligand property	Orthosteric (matched)	Allosteric (matched)	p-value (after matching)
**Molecular weight (Da)**	385 ± 52	382 ± 55	0.41
**LogP**	2.8 ± 1.1	2.7 ± 1.2	0.38
**Rotatable bonds**	4.2 ± 1.8	4.1 ± 1.9	0.52
**H-bond donors**	1.5 ± 0.9	1.4 ± 1.0	0.44
**H-bond acceptors**	4.1 ± 1.5	4.0 ± 1.6	0.47
**Performance (AF3)**			
**Ligand RMSD (Å)**	2.5 ± 0.5	6.1 ± 1.7	<0.001
**Success @2Å (%)**	78	27	<0.001

*Notes: Propensity score matching was performed on the five ligand properties listed above, yielding 502 matched pairs with no significant differences in any property. Performance metrics are reported for AF3 oracle predictions.

Table S10. Stratification by apo-holo conformational change magnitude (Cα RMSD)

Conformational change	Site type	Ligand RMSD (Å)	Success @2Å (%)
**Small (<1.0 Å)**	Orthosteric	2.2 ± 0.4	83
	Allosteric	5.4 ± 1.3	32
**Medium (1.0–2.0 Å)**	Orthosteric	2.5 ± 0.6	75
	Allosteric	6.0 ± 1.5	27
**Large (>2.0 Å)**	Orthosteric	2.8 ± 0.7	67
	Allosteric	6.5 ± 1.8	23

Table S11. Bootstrap subsampling to match dataset size.*

Subsampling iteration	Orthosteric RMSD (mean ± SD)	Allosteric RMSD (mean ± SD)	Gap	p-value
**1**	2.7 ± 0.4	6.1 ± 1.5	3.4 Å	<0.001
**2**	2.6 ± 0.5	5.9 ± 1.6	3.3 Å	<0.001
**Median over 1,000 iterations**	2.7 (range 2.5–2.9)	6.0 (range 5.4–6.2)	3.3 Å (range 2.9–3.7)	all <0.001

*Notes: The orthosteric dataset (originally 2,275 complexes) was randomly subsampled to match the size of the allosteric dataset (1,966 complexes). One thousand bootstrap subsamples were drawn. In every single iteration, the mean orthosteric ligand RMSD remained below 3.0 Å, the mean allosteric RMSD remained above 5.4 Å, and the difference was highly significant ($p < 0.001$, Mann-Whitney U test).

Table S12. Conformational frustration distributions for allosteric complexes in the ASD-derived test set (182 unique sites).*

State	Region	Minimally frustrated (MF)	Neutrally frustrated (NF)	Highly frustrated (HF)
**Apo**	Global fold	48.2% (±4.1)	34.5% (±3.8)	17.3% (±2.9)
	Binding site	22.1% (±5.2)	71.4% (±6.1)	6.5% (±2.3)
**Holo**	Global fold	47.9% (±4.3)	34.8% (±3.9)	17.3% (±3.0)
	Binding site	23.5% (±5.0)	69.8% (±5.8)	6.7% (±2.4)

*Notes: Values are mean ± SD over all complexes. Conformational frustration Z-score thresholds: MF ($Z > 0.78$), NF ($-1.0 \leq Z \leq 0.78$), HF ($Z < -1.0$). Global fold includes all residues; binding site defined as residues within 4 Å of the ligand. Neutral frustration dominates binding sites in both apo and holo states (≈70–71%), with no significant change upon ligand binding. Minimal frustration remains low (≈22–24%), and highly frustrated contacts are rare (≈6–7%). These distributions are statistically indistinguishable from those of the original allosteric benchmark ($p > 0.3$, Mann-Whitney U test).

Table S13. Conformational frustration distributions for allosteric complexes in the non-redundant PLA set (182 unique pockets).*

State	Region	Minimally frustrated (MF)	Neutrally frustrated (NF)	Highly frustrated (HF)
**Apo**	Global fold	49.1% (±4.5)	33.8% (±4.0)	17.1% (±3.1)
	Binding site	21.5% (±5.4)	72.2% (±6.3)	6.3% (±2.2)
**Holo**	Global fold	48.8% (±4.4)	34.1% (±4.1)	17.1% (±3.0)
	Binding site	22.9% (±5.1)	70.5% (±5.9)	6.6% (±2.3)

*Notes: Values are mean ± SD. The same thresholds and definitions apply. Again, neutral frustration dominates allosteric binding sites (≈70–72% in both states), with no systematic frustration quenching upon ligand binding. Minimal frustration remains below 23%, and highly frustrated contacts are rare. These results are virtually identical to those from the ASD dataset and the original allosteric benchmark, confirming the robustness of the neutral frustration signature across independent allosteric datasets.

Table S14. Mutational frustration distributions for allosteric complexes in the ASD-derived test set.*

State	Region	Minimally frustrated (MF)	Neutrally frustrated (NF)	Highly frustrated (HF)
**Apo**	Global fold	28.5% (±3.6)	52.3% (±4.8)	19.2% (±3.2)
	Binding site	31.2% (±5.5)	58.4% (±6.2)	10.4% (±2.8)
**Holo**	Global fold	28.3% (±3.5)	52.5% (±4.7)	19.2% (±3.1)
	Binding site	32.1% (±5.3)	57.6% (±6.0)	10.3% (±2.7)

*Notes: Mutational frustration quantifies evolutionary optimality. Thresholds: MF ($Z > 0.78$), NF ($-1.0 \le Z \le 0.78$), HF ($Z < -1.0$). Neutral mutational frustration dominates allosteric binding sites (≈58%), reflecting evolutionary permissiveness. Minimal frustration is modest (≈31–32%), and highly frustrated contacts are relatively low (≈10%). There is no significant change from apo to holo. This pattern is consistent with the original allosteric benchmark.

Table S15. Mutational frustration distributions for allosteric complexes in the non-redundant PLA set.*

State	Region	Minimally frustrated (MF)	Neutrally frustrated (NF)	Highly frustrated (HF)
**Apo**	Global fold	29.1% (±3.8)	51.8% (±4.9)	19.1% (±3.3)
	Binding site	30.5% (±5.6)	59.2% (±6.4)	10.3% (±2.9)
**Holo**	Global fold	28.9% (±3.7)	52.0% (±4.8)	19.1% (±3.2)
	Binding site	31.3% (±5.4)	58.4% (±6.1)	10.3% (±2.8)

*Notes: Neutral mutational frustration dominates allosteric binding sites (≈58–59%), with no significant change upon ligand binding. Minimal frustration remains around 31%. These values are highly consistent with the ASD dataset and the original allosteric benchmark, confirming that the evolutionary permissiveness of allosteric sites is a general feature.

Table S16. Correlation between per-residue minimal frustration density and model confidence for orthosteric and allosteric complexes

Model	Dataset	Site type	Spearman ρ	95% CI	p-value
**AF3**	Original	Orthosteric	0.52	[0.48, 0.56]	<0.001
	Original	Allosteric	0.08	[0.02, 0.14]	0.12
	ASD	Allosteric	0.07	[0.00, 0.14]	0.18
	PLA (non-red)	Allosteric	0.09	[0.02, 0.16]	0.10
**Protenix**	Original	Orthosteric	0.48	[0.44, 0.52]	<0.001
	Original	Allosteric	0.06	[−0.01, 0.13]	0.24
**Chai-1**	Original	Orthosteric	0.55	[0.51, 0.59]	<0.001
	Original	Allosteric	0.12	[0.05, 0.19]	0.03
**Boltz-2**	Original	Orthosteric	0.44	[0.40, 0.48]	<0.001
	Original	Allosteric	0.05	[−0.02, 0.12]	0.31
**DynamicBind**	Original	Orthosteric	0.46	[0.42, 0.50]	<0.001
	Original	Allosteric	0.10	[0.03, 0.17]	0.07
